# Adamantyl Side Chains as Anti‐Aggregating Moieties in Dyes for Dye‐Sensitized Solar Cells

**DOI:** 10.1002/chem.202201726

**Published:** 2022-07-20

**Authors:** David Moe Almenningen, Brita Susanne Haga, Henrik Erring Hansen, Audun Formo Buene, Bård Helge Hoff, Odd Reidar Gautun

**Affiliations:** ^1^ Department of Chemistry Norwegian University of Science and Technology Høgskoleringen 5 7491 Trondheim Norway; ^2^ Department of Materials Science and Engineering Norwegian University of Science and Technology Sem Sælands vei 12 7491 Trondheim Norway; ^3^ Department of Civil and Environmental Engineering Norwegian University of Science and Technology Høgskoleringen 7a 7034 Trondheim Norway

**Keywords:** adamantane, anti-aggregation, anti-recombination, dye design, dye-sensitized solar cells, triarylamine dye

## Abstract

Designing and evaluating novel dye concepts is crucial for the development of the field of dye‐sensitized solar cells (DSSCs). In our recent report, the novel concept of tethering the anti‐aggregation additive chenodeoxycholic acid (CDCA) to dyes for DSSC was introduced. Based on the performance improvements seen for this modification, the aim of this study is to see if a simplified anti‐aggregation unit could achieve similar results. The following study reports the synthesis and photovoltaic characterization of two novel dyes decorated with the steric ethyladamantyl moiety on the π‐spacer, and on the triarylamine donor. This modification is demonstrated to be successful in increasing the photovoltages in devices employing copper‐based electrolytes compared to the non‐modified reference dye. The best photovoltaic performance is achieved by a device prepared with the adamantyl decorated donor dye and CDCA, this device achieves a power conversion efficiency of 6.1 % (Short‐circuit current=8.3 mA cm^−2^, Open‐circuit voltage=1054 mV, Fill factor=0.69). The improved photovoltaic performance seen for the adamantyl decorated donor demonstrate the potential of ethyladamantyl side chains as a tool to ensure surface protection of TiO_2_.

## Graphical abstract

**Two novel triarylamine** dyes are reported, that are decorated with ethyladamantyl side chains on the π‐spacer (**BSH‐2**), and on the donor (**BSH‐3**). The ethyladamantyl modified dyes display higher photovoltages than the non‐modified reference dye (**JK‐305**). DSSC devices prepared with the adamantyl decorated donor dye (**BSH‐3**) and the anti‐aggregation additive CDCA display the best PCE of the series, at 6.1 %.

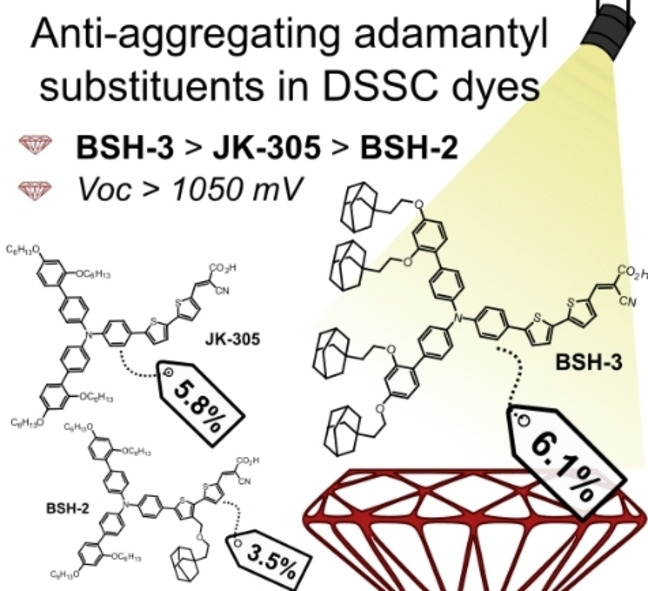

## Introduction

The dye‐sensitized solar cell (DSSC), a technology that recently turned 30 years since its original report in 1991, has shown remarkable promise in recent years for ambient light photovoltaics.[^1^, ^2^] The typical device consists of three key components: a layer of mesoporous semiconducting metal‐oxide (most commonly TiO_2_), a light‐absorbing dye which is attached on the metal‐oxide, and a redox mediator responsible for regenerating the oxidized sensitizers.^[3]^ Owing to this multifaceted nature, where optimization of the devices can be achieved through tuning of the separate parts, the technology still remains relevant and under constant development today. The introduction of novel copper‐based redox mediators where the energy levels of the electrolyte (0.87–0.97 V vs. the standard hydrogen electrode (SHE)) are more closely matched to the energy levels of the dyes, has led to a drastic reduction in overpotential losses of the devices compared to the traditional I^−^/I_3_^−^‐electrolyte (0.5 V vs. SHE).^[4]^ These new mediators have enabled devices with power conversion efficiencies (PCE) of more than 13 % under standard AM1.5G sunlight, and efficiencies surpassing 30 % under lowlight conditions.[^5^, ^6^] The development of novel dyes displaying photochromic behavior,^[7]^ and dyes displaying near‐infrared (NIR) absorption facilitate devices with tunable transparency.^[8]^ This property lends itself nicely for the use of DSSC in building integrated photovoltaics (BIPV), where transparency of the devices is important to make them visually non‐intrusive.[^9^, ^10^]

**Figure 1:**
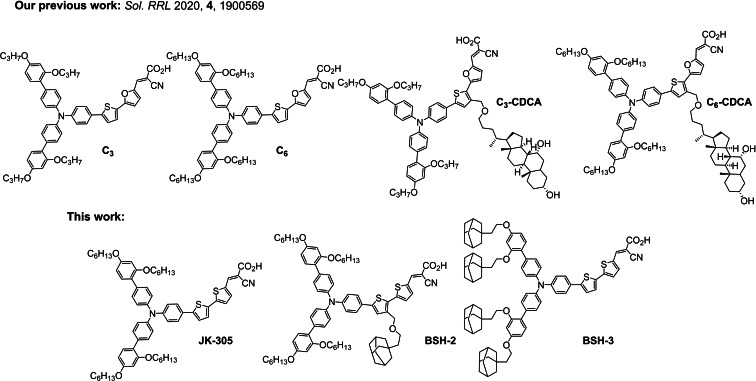
The sensitizers prepared in our previous work represented the first report of the common anti‐aggregation additive CDCA tethered to a dye covalently. The dyes prepared in study is the first report of ethyladamantyl as side chains in dyes for DSSC.

Fully organic dyes offer a unique opportunity to tailor optical and or electronical properties on a molecular level. The absorption properties of dyes that adopt a donor – π‐spacer – acceptor (D‐π‐A) motif is easily modified by varying the acceptor or donor strength of the building blocks, or through modification of the π‐conjugated linker.^[11]^ Expanding the π‐conjugated system broadens the spectral response of dyes, and increases the attainable short‐circuit current of the solar cells.[^12^, ^13^, ^14^] However, such an expansion of the aromatic system leaves the dyes susceptible to aggregate on the surface of TiO_2_. This blueshifts the absorption of the dyes and facilitates quenching of the excited states of the dyes.^[15]^ Implementing measures to combat the undesirable aggregation effects has been a topic for many studies over the years and a wide variety of approaches has been designed and evaluated. Linking multiple dyes together has proven valuable in reducing aggregation,[^16^, ^17^, ^18^] optimizing the bulkiness of the sidechains on the aromatic system is another proven method.[^19^, ^20^, ^21^] It is also quite common to add anti‐aggregation additives to the staining solution, the most common additive for DSSC is chenodeoxycholic acid (CDCA).^[22]^ In our previous study on anti‐aggregation measures for DSSC, see Figure 1, we prepared two dyes with CDCA tethered to their π‐spacer (**C_3_‐CDCA** and **C_6_‐CDCA**), and found this to be beneficial for photovoltaic performance compared to the reference dyes (**C_3_** and **C_6_**).^[23]^ Our previous study also highlighted the anti‐aggregational effect of the alkoxy chains on the donor side of the molecules, where superior performance was seen for the dyes with longer side chains (**C_6_** and **C_6_‐CDCA** vs. **C_3_** and **C_3_‐CDCA)**. Some of the improvements seen for this structural modification is likely due to another closely related aspect of solar cell performance, namely the recombination of electrons in TiO_2_ with the oxidized species of the electrolyte. This loss process is known to lower the Fermi level of TiO_2_, effectively reducing the attainable open‐circuit voltage (*V_oc_*) of the dye‐sensitized solar cell.^[24]^ Ever since moving to one‐electron redox mediators, such as Co^2+^/Co^3+^ and Cu^+^/ Cu^2+^ complexes, the retardation of recombination has been a key goal for molecular engineering of novel dyes, as Co^3+^ and Cu^2+^ species of the novel electrolytes undergo a more rapid recombination with electrons in TiO_2_ compared to the conventional I_3_^−^‐complex.[^25^, ^26^, ^27^] The dye **MS5**, a striking example of successful molecular engineering to circumvent the effect of recombination, was presented in a recent paper by Zhang et al.^[6]^ This dye employs dodecyloxy side chains that suppresses recombination expertly to allow for copper‐based devices with a record photovoltage of 1.24 V.

With the promising results from our initial study of tethering the highly steric CDCA on the π‐spacer, we set out to investigate if a simplified blocking group could achieve similar performance improvements. It has been shown that introducing too large side chains is bad for overall photovoltaic performance.^[20]^ We therefore prepared a dye (**BSH‐2**), shown in Figure 1, which had 1‐ethyladamantyl tethered to the π‐spacer in an analogous manner to the previously reported CDCA‐dyes. Adamantane is a rather unique hydrocarbon consisting of three fused cyclohexane rings in a rigid but strain‐free ring system.^[28]^ Adamantyl substitutions are common structural motifs in the field of antiviral pharmaceuticals.[^29^, ^30^, ^31^, ^32^] Besides this, adamantyl's unique properties have also been utilized in organic optoelectronic materials such as organic hole‐transporting materials (HTM),[^33^, ^34^] or as a side chain on diketopyrrolopyrrole (DPP) pigments.[^35^, ^36^] In the field of DSSC we have also seen some use of adamantane derivatives, where 1‐admantane acetic acid (ADAA) has been evaluated as an anti‐aggregation additive,^[22]^ the nitroxide radical 2‐azaadamantan‐*N*‐oxyl (AZA) has been employed as a redox mediator,^[37]^ and most notably AZA has found use in a tandem redox system with a Co^2+/3+^ complex to achieve *V_oc_* values above 1 V.^[38]^ With the illustrious properties of the dodecyloxy substituted donor of **MS5** in mind, we also set out to prepare a dye (**BSH‐3**), shown in Figure 1, which is fitted with adamantylethyloxy (another 12‐carbon moiety) substituents on the triarylamine donor. The effects of the ethyladamantyl substituents on **BSH‐2** and **BSH‐3** will be compared with the performance of the reference dye **JK‐305**, a standard triarylamine dye bearing hexyloxy side chains and a 2’,2‐bithiophene π‐spacer. The dye **JK‐305**, displayed a PCE of 7.3 % (*J_sc_* =13.1 mA cm^−2^, *V_oc_* =760 mV, FF=0.73) in an iodine‐based electrolyte.^[39]^ The results presented will serve as a model study to illustrate the potential merits, and eventual pitfalls of the steric ethyladamantyl substitutions on the π‐spacer and on the donor of dyes for DSSC.

## Results and Discussion

### Dye synthesis

**Scheme 1:**
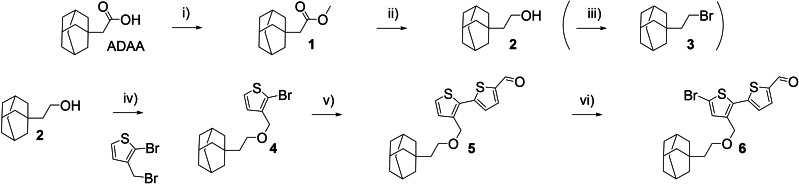
Synthesis of π‐spacer building block **6**, and key intermediate **3** used in the synthesis of the adamantyl‐donor. i) MeOH, H_2_SO_4_, r.t., 79 %, ii) LiAlH_4_, THF, r.t., 79 %, iii) HBr, 100 °C, 94 %, iv) NaH, NaI THF, 50 °C, 75 % v) (5‐formylthiophene‐2‐yl)boronic acid, XPhos Pd G3, K_3_PO_4_, H_2_O/1,4‐dioxane, 80 °C, 54 %, vi) NBS, CH_2_Cl_2_, 0 °C, 66 %.

The synthesis of the dyes is based on the route used for the CDCA‐dyes previously reported by our group.^[23]^ However, in contrast to CDCA, the adamantyl‐moiety bears no additional heteroatoms and this simplified the synthesis somewhat as it made protection chemistry redundant. The preparation of the adamantyl‐functionalized π‐spacer is shown in Scheme 1, starting from the commercially available adamantyl acetic acid (ADAA). The acid was subjected to a Fischer esterification, yielding the methyl ester **1** and a reduction giving the adamantyl ethanol **2**. Compound **2** was used to prepare the bromide **3** needed for the synthesis of the adamantyl decorated donor, which will be discussed later. Further, the alcohol **2** was fused with bromo‐3‐(bromomethyl)thiophene using a Williamson ether synthesis under Finkelstein conditions, to produce **4**. A Suzuki–Miyaura cross‐coupling catalyzed by XPhos Pd G3 reported by Bruno et al.^[40]^ was used to produce bithiophene **5**. A final bromination using NBS yielded the finished π‐spacer building block **6**.

**Scheme 2:**
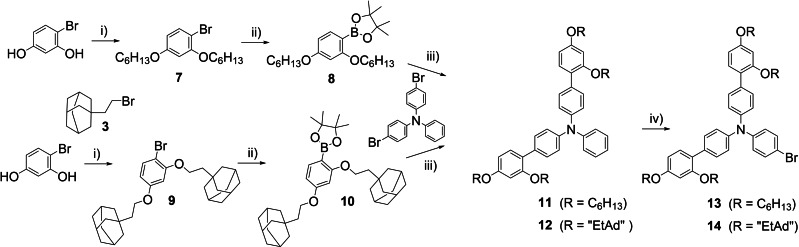
Synthesis of key donor intermediates **13** and **14**. i) KOH, DMSO, 1‐bromohexane/intermediate **3**, r.t., 82 %/49 %, ii) HBpin, PdCl_2_(CH_3_CN)_2_, SPhos, Et_3_N/1,4‐dioxane, 110 °C, iii) Pd(OAc)_2_, SPhos, K_2_CO_3_, H_2_O/1,4‐dioxane, 80 °C, 87 %/56 %, iv) NBS, CH_2_Cl_2_, r.t., 99 %/95 %.

The synthesis of the adamantyl donor followed our previously reported synthesis of triarylamine donors, shown in Scheme 2. Interestingly, the Williamson ether synthesis involving the adamantyl bromide **3** suffered from a competing elimination reaction. As a result of this, the yield of **9** was 49 % which is considerably lower than the yield of **7** at 82 %. The subsequent Pd‐catalyzed borylation reaction reported by Billingsley and Buchwald,^[41]^ showed full conversion to the corresponding boronates **8** and **10**. A Suzuki–Miyaura reaction of 4,4’‐dibromotriphenylamine yielded the functionalized triarylamines **11** and **12**, in yields of 87 % and 56 %, respectively. The ultimate step in the donor synthesis was a regiospecific bromination using NBS, where **13** and **14** were prepared in yields of 99 % and 95 %, respectively.

**Scheme 3:**
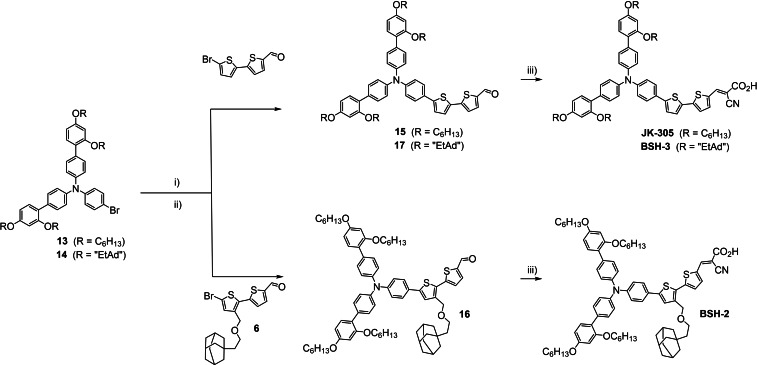
Synthesis route for the merging of donor and π‐spacer moieties and preparation of dyes **JK‐305**, **BSH‐2**, and **BSH‐3**. i) HBpin, PdCl_2_(CH_3_CN)_2_, SPhos, Et_3_N/1,4‐dioxane, 110 °C, ii) Pd(OAc)_2_, SPhos, 1,4‐dioxane/H_2_O, 80 °C, 43 %(**15**)/48 %(**16**)/29 %(**17**), iii) cyanoacetic acid, piperidine, CH_3_CN, 80 °C, 79 %(**JK‐305**)/67 %(**BSH‐2**)/57 %(**BSH‐3**).

The π‐spacer and the donor moieties were merged, and the dyes completed as shown in Scheme 3. Firstly, the brominated triarylamines were converted to the corresponding boronic esters using the Pd‐catalyzed borylation reaction. The crude boronate products were then used in a Suzuki–Miyaura cross coupling, yielding the dye precursors **15**–**17** in yields of 29–48 % over two steps from the brominated triarylamine. The final step was a Knoevenagel condensation, introducing the cyano acetic acid anchoring group giving the sensitizers **JK‐305**, **BSH‐2**, and **BSH‐3** in yields of 57–79 %.

### Photophysical properties

**Figure 2:**
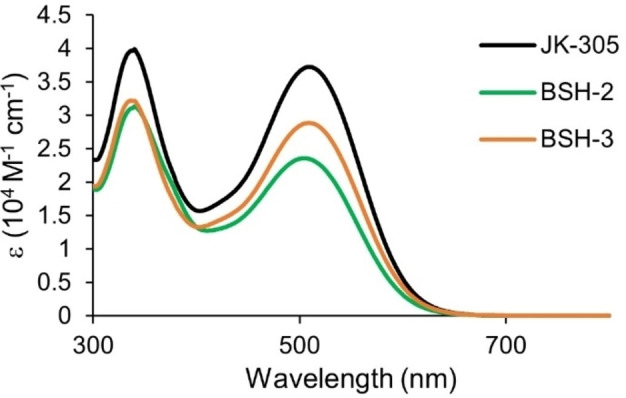
Absorption spectra of all dyes in dichloromethane solution.

**Figure 3:**
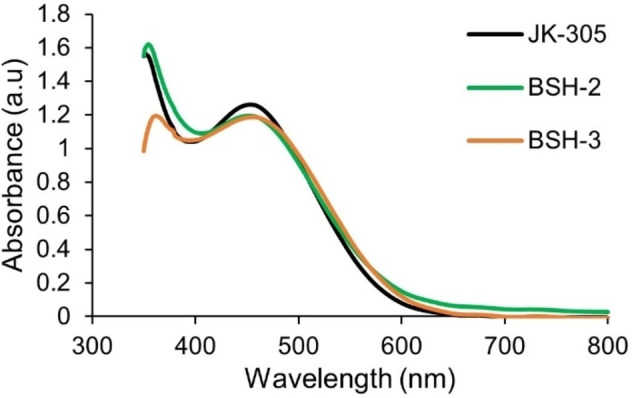
UV/vis measurements of all dyes on TiO_2_ films (2.5 μm, 18NR‐T, Greatcell Solar).

The photophysical properties of dyes are essential for their performance as sensitizers in dye‐sensitized solar cells. It is crucial that modifications done on the dye molecules does not adversely affect their absorption properties in solution or when attached on TiO_2_. UV/Vis measurements of the dyes were performed on the dyes in dichloromethane solution (2 ⋅ 10^−5^ m), and adsorbed on a TiO_2_ film (2.5 μm, 18NR‐T, Greatcell Solar). The results from these measurements are shown in Figure 2, and Figure 3 and are summarized in Table 1.

**Table 1:** Photophysical and electrochemical properties of dyes in the series.

Dye	λ_abs_^[a]^ [nm]	ϵ [m ^−1^cm^−1^]	Em.^[b]^ [nm]	λ_abs_^[c]^ on TiO_2_ [nm]	E_0‐0_^[d]^ [eV]	E_ox_^[e]^ [V vs. SHE]	E_LUMO_^[f]^ [V vs. SHE]
**JK‐305**	510	37300	632	454	2.12	1.10	−1.02
**BSH‐2**	505	23600	629	453	2.19	1.11	−1.08
**BSH‐3**	509	28900	634	456	2.13	1.14	−0.99

[a] Maximum of most redshifted peak measured in dichloromethane solution (2 ⋅ 10^−5^ m). [b] Emission when ICT band is excited, in dichloromethane solution. [c] Maximum of most redshifted peak on TiO_2_ (2.5 μm, GreatcellSolar 18NR‐T). [d] Calculated from the intersection of the absorption and normalized emission spectra. [e] Measured vs. F_c_^+^/F_c_ on stained TiO_2_ electrodes in acetonitrile with 0.1 M LiTFSI, converted to V vs. SHE by 0.624 V. Scan rate 10 mV s^−1^. [f] Calculated from E_ox_‐E_0‐0_.

Comparing the data obtained from our measurements with the previously reported data of the dye **JK‐305**, a solvatochromic effect on the absorption properties is seen.^[39]^ The absorption maximum is found at 510 nm in dichloromethane, a redshift of 59 nm compared to the previously reported data in ethanol. The optical band gap is also found to be smaller from the measurements done in dichloromethane (2.12 eV) compared to the measurements in ethanol (2.30 eV). The optical properties of the adamantyl‐functionalized dyes in solution are similar to the reference dye **JK‐305**, where the absorption maxima are found within 5 nm of each other. However, the molar extinction coefficient of **JK‐305** is approximately 9000–13000 m ^−1^ cm^−1^ higher than that of **BSH‐2** and **BSH‐3**. The slightly bigger optical band gap of **BSH‐2** could suggest that the adamantyl‐moiety forces a slight out‐of‐plane ring twist between the two thiophenes, as previous quantum calculations has demonstrated that side chain modified thiophenes display larger dihedral angles than non‐modified ones.^[42]^ The absorption properties of the dyes on TiO_2_ films are also remarkably similar, where a 52–56 nm blueshift of absorption maxima is seen for the dyes in this series when adsorbed on TiO_2_. This blueshift is frequently attributed to a combination of deprotonation of the carboxylic acid, and formation of H‐aggregated dye clusters on the TiO_2_ surface.[^43^, ^44^]

When comparing **JK‐305** to our previously reported dye **C_6_**, which is entirely similar except for a thiophene‐furan π‐spacer instead, we see that absorption maximum is redshifted by 8 nm and the intensity of absorption is increased by 10 000 m ^−1^ cm^−1^. When we introduced the CDCA‐moiety on the π‐spacer of **C_6_‐CDCA** we saw a blueshift of 13 nm compared to the reference dye, while the introduction of adamantyl on the π‐spacer of **BSH‐2** blueshifted absorption by 5 nm. Introduction of adamantyl on the donor of **BSH‐3**, had negligible effect on the absorption maximum compared to the reference dye. This shows that the adamantyl‐functionalization is even more useful in terms of absorption properties than the CDCA‐functionalization in our previous study.

### Electrochemical properties

**Figure 4:**
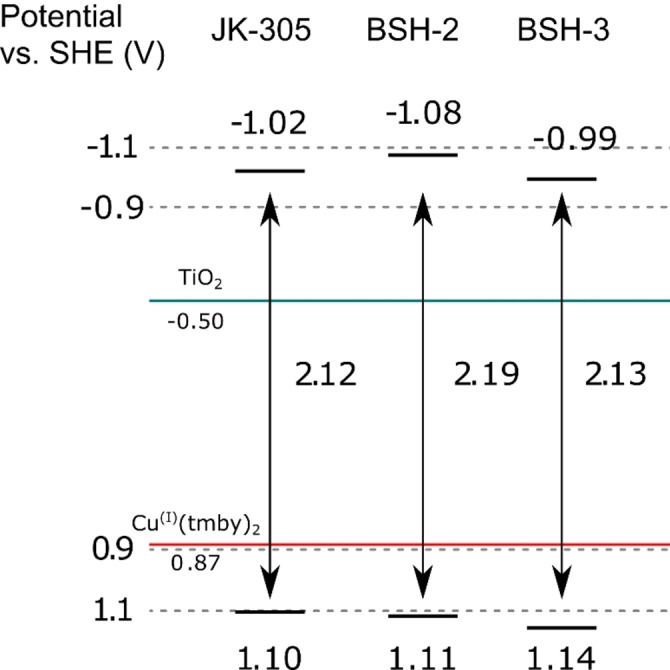
Energy levels of the frontier orbitals for the sensitizers in this study.

Another crucial performance parameter for the sensitizers in dye‐sensitized solar cells is their electronic properties. The HOMO energy level of the dye should leave sufficient driving force for the oxidized dye to be regenerated by the redox shuttle. Additionally, the excited state energy level should have a sufficient driving force for electron injection into the metal oxide. The oxidation potentials of the dyes were found through cyclic voltammetry (CV) experiments of the dyes sensitized on TiO_2_ films. The LUMO level was calculated by subtracting the optical band gap from the measured oxidation potential. The energy levels of the frontier orbitals of the dyes in relation to the electrolyte and the conduction band of TiO_2_ is shown in Figure 4.

All the dyes displayed reversible oxidation peaks around 1.10–1.14 V versus SHE, suggesting that the modifications done on the dyes had minor effect on their frontier orbitals. This is also seen in our previous publication, where the CDCA‐moiety had little or no effect on the oxidation potential of the dye. The CV measurements revealed that all the dyes would be compatible for use in a device with a Cu^+^/Cu^2+^ redox shuttle, as the HOMO energy levels are sufficiently deep. The dye which displayed the highest oxidation potential of 1.10 V versus SHE (**JK‐305**), still had a driving force of 0.23 V which is more than the 0.1 V previously established to be sufficient.^[4]^

### Photovoltaic properties

**Figure 5:**
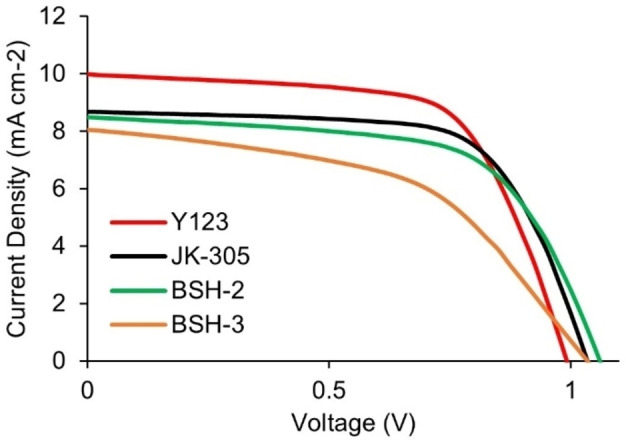
J‐V curves for the best parallel of each dye in devices without anti‐aggregation additives, obtained under 1 sun AM 1.5G illumination. Also included is the benchmark dye **Y123** with 4 equiv. CDCA.

**Figure 6:**
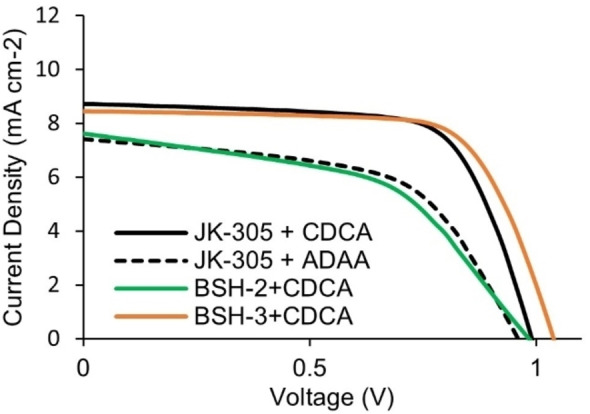
J‐V curves for the best parallel of each dye in devices with anti‐aggregation additives, obtained under 1 sun AM 1.5G illumination.

The fabrication and characterization of DSSC‐devices sensitized with the dyes, revealed that insertion of adamantyl‐groups was beneficial for increasing the *V_oc_* of the devices. The data from the photovoltaic characterization is summarized in Table 2, the data presented is the average values obtained from three separate devices. The *J*‐*V* curves of the best performing devices from each parallel are shown in Figure 5 and Figure 6. In our recent review on the class of phenothiazine dyes, we saw that one dye (**H‐PTZ**) had been investigated in 13 separate papers with PCE values ranging from 3.1 % to 6.6 %.^[45]^ This showed us the value of using known reference sensitizers to provide a benchmark for the findings presented in a study. In this study we prepared devices sensitized with the known dye **Y123** to serve as a reference, the efficiency of the **Y123** solar cell was 6.5 % (*J_sc_* =10.0 mA cm^−2^, *V_oc_* =992 mV, FF=0.65).

**Table 2:** Photovoltaic performance of all dyes under 1 sun AM 1.5G illumination, and from IPCE measurements. Results from dye loading experiments are also included.

Dye	Additive [10 equiv.]	IPCE *J_SC_* [mA cm^−2^]^[a]^	*J_SC_* [mA cm^−2^]	*V_OC_* [mV]	FF	PCE [%]	Dye Loading [10^−8^ mol cm^−2^]^[b]^
JK‐305	–	8.94	8.6±0.1	1035±4	0.64±0.02	5.7±0.2	11.2±0.1
	CDCA	8.32	8.4±0.3 ^*c*^	972±19 ^*c*^	0.70±0.01^[c]^	5.8±0.2^[c]^	9.0±0.5
	ADAA	7.83	7.5±0.2	955±4	0.55±0.03	3.9±0.2	10.8±0.03
BSH‐2	–	8.24	8.2±0.2	1067±5	0.63±0.01	5.5±0.1	8.6±0.02
	CDCA	6.11	7.4±0.2	975±8	0.49±0.03	3.5±0.2	6.8±0.3
BSH‐3	–	8.84	7.9±0.2	1045±3	0.45±0.04	3.7±0.3	8.4±0.03
	CDCA	8.38	8.3±0.4	1054±14	0.69±0.01	6.1±0.2	6.8±0.2
Y123^[d]^	CDCA (4 equiv.)	9.92	10.0	992	0.65	6.5	8.2±0.2

[a] Obtained by integration of the IPCE spectrum over the 1 sun AM 1.5 G spectrum. [b] Values averaged of two desorbed TiO_2_‐electrodes. [c] Average values of two cells. [d] Values from best‐performing device.

When comparing the performance of the devices without any additives in the staining solution the best performing dye was the reference dye **JK‐305** (5.7 %), narrowly outperforming the dye with a modified π‐spacer, **BSH‐2** (5.5 %). The dye with a modified donor, **BSH‐3**, displayed a worse performance under these conditions owing to a remarkably low fill factor of 45 %. It should be noted, however, that the open‐circuit voltages of all three dyes under these conditions exceeded 1.0 V which was fairly promising with regards to the dyes’ ability to insulate the surface of TiO_2_ and prevent recombination.

In our previous work we saw that all the dyes, even the CDCA‐substituted dyes, benefitted from 10 equivalents of additional CDCA added in the staining solution. With this in mind we set out to prepare a set of new devices with 10 equivalents of CDCA added in the staining solution. For the reference dye **JK‐305**, a slight improvement in PCE was seen, mainly due to an improved fill factor of the devices. The devices prepared with **BSH‐2** and CDCA revealed a surprising outcome, where the additive reduced the overall performance of the device significantly. It is the only dye in this and the previous study which has not benefitted from the addition of CDCA. This could suggest that there are some undesirable interactions between the adamantyl‐substituents and CDCA occurring on the surface of TiO_2_. Furthermore, the adamantyl‐donor dye, **BSH‐3**, benefitted immensely from addition of CDCA, and improvement of all performance parameters was seen. The most pronounced effect was seen for the fill factor which increased from 45 % to 69 %. As a consequence of this drastic improvement, the **BSH‐3**/CDCA combination was the most efficient device in the series (*J_sc_* =8.3 mA cm^−2^, *V_oc_* =1054 mV, FF=0.69, PCE=6.1 %). This shows that the adamantyl groups could offer an improvement over the conventional hexyl chains, due to the higher photovoltages that this modification allows.

The excellent photovoltage of **BSH‐2** (1.07 V) led us to wonder whether adamantyl acetic acid (ADAA) introduced in the staining solution could work as an anti‐aggregation additive. Therefore, we prepared a set of devices where we added the same amount of ADAA (10 equiv.) to the staining solution of **JK‐305**. The devices prepared with ADAA saw a reduction in all performance parameters. Excluding **BSH‐3**, the devices fabricated with additives produce lower photovoltages in all cases. This can be explained by increased protonation that lowers the Fermi level of TiO_2_.^[46]^ The deleterious interactions between dye and additive seen for the **BSH‐2**/CDCA and **JK‐305**/ADAA might also facilitate recombination which would also explain the lower photovoltage observed. When considering the dye loading experiments, the addition of CDCA reduced dye loading by ≈20 % in all three cases. The addition of ADAA reduced dye loading by only 4 %. In addition, the reduced fill factor and *J_sc_* seen for the devices prepared with **JK‐305** and ADAA suggest that this additive has no anti‐aggregation effect. When combining this with the adverse effect that increased protonation has on the photovoltage, we do not recommend adding ADAA to future staining solutions. In a previous study on the anti‐aggregation effect of ADAA, a similar reduction of photovoltage was seen.^[22]^

**Figure 7:**
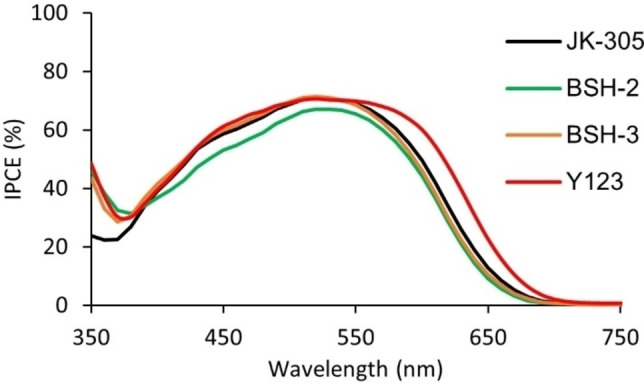
IPCE spectra of the best performing parallel for each dye.

**Figure 8:**
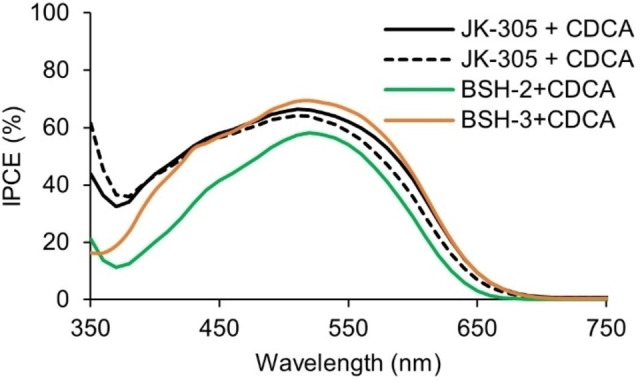
IPCE spectra of the best performing parallel for each dye.

The IPCE‐spectra of the devices fabricated in this study are shown in Figure 7 and Figure 8. The maxima for all the dyes are found at 60–70 % which shows that the devices suffer from suboptimal light harvesting. It seems that the molar extinction coefficient is not the only cause for the limited light harvesting ability, as **Y123** (ϵ=48000 m ^−1^ cm^−1^) and **BSH‐3** (ϵ=28900 m ^−1^ cm^−1^) both display similar maxima of around 70 %.

The effect of the adamantyl modifications was also apparent when considering the results of the dye loading experiments. We see that the ethyladamantyl substitutions lower the dye loading by ≈25 % compared to the reference dye, independently of added anti‐aggregation additives. It also shows that one substituent on the π‐spacer lowers the dye loading by the same amount as four substituents on the donor. The dye **BSH‐2** display a considerably higher dye loading (6.8 ⋅ 10^−8^ mol cm^−2^), than its CDCA‐substituted analogue **C_6_‐CDCA** (4.2 ⋅ 10^−8^ mol cm^−2^) under similar conditions. This suggest that the more compact adamantyl substituent has a smaller steric impact than the CDCA‐substituent.

### Electrochemical impedance spectroscopy

**Figure 9:**
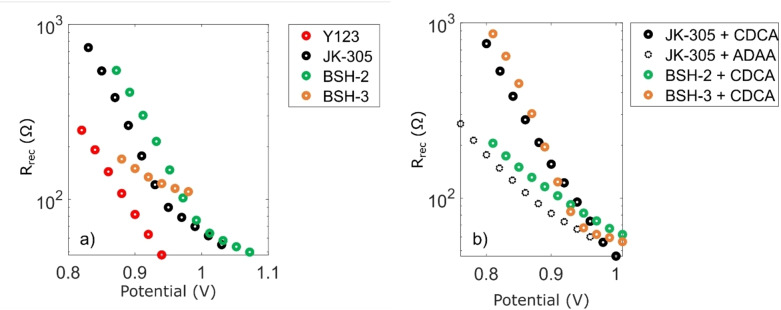
The recombination resistances of the dyes, obtained from EIS under monochromatic light, plotted against applied voltage for left) the devices without anti‐aggregation additives and the benchmark dye **Y123**, right) the devices with anti‐aggregation additives.

To further evaluate the anti‐recombination properties of the adamantyl moieties we performed impedance spectroscopy experiments on the different devices. The complex plane plots of the devices are shown in Figure S3 in the Supporting Information, and the recombination resistance as a function of applied voltage is shown in Figure 9. In the devices prepared without any additives, **JK‐305** and **BSH‐2** display higher recombination resistances than the adamantyl donor dye **BSH‐3**. This is in stark contrast to the devices prepared with additives, where **BSH‐3** display marginally the highest recombination resistance in the series. This further highlights the benefit of added CDCA in staining solution to improve device performance through surface passivation in addition to its anti‐aggregational effect.^[47]^ Meanwhile, the addition of CDCA to **BSH‐2** resulted in a facilitation of recombination which could stem from undesired interactions between the adamantyl on the π‐spacer and CDCA. This is in contrast to the results from our previous study, where the CDCA‐substituted π‐spacer dyes showed improved electron lifetimes from addition of CDCA to the staining solution. The use of ADAA as an additive in the staining solution of **JK‐305** also proved to be detrimental, as it lowered the recombination resistance compared to the additive‐free **JK‐305** device, and the **JK‐305**/CDCA device. The results from EIS support the hypothesis that the lower photovoltage measured for devices prepared with anti‐aggregation additives is due to a facilitation of recombination.

## Conclusion

By introducing ethyladamantyl substitutions on two different locations of the triarylamine dye scaffold, namely the π‐spacer and the donor, we designed and synthesized two novel dyes **BSH‐2** and **BSH‐3**. The effects of these substitutions were evaluated by comparing them to the performance of a reference dye without any modifications, the previously reported **JK‐305** dye. Overall, we saw that the ethyladamantyl substituted dyes produced higher photovoltages than the reference dye in all cases. The photovoltaic performance of the dye with an adamantyl‐substituted π‐spacer, **BSH‐2**, showed sign of deleterious interactions between the substituent and CDCA. The EIS measurements revealed that the **BSH‐2**/CDCA device suffered from a lower recombination resistance compared to the additive‐free **BSH‐2** device. Conversely, the adamantyl donor dye, **BSH‐3**, got a significant boost in photovoltaic performance from added CDCA, EIS measurements showed that CDCA had increased the recombination resistance considerably. The best photovoltaic performance, and the highest recombination resistance was seen from the **BSH‐3**/CDCA device, which suggest that this donor could be of use for future dye designs.

## Experimental Section

**Materials and reagents**: All reactions were performed under inert N_2_‐atmosphere. The chemicals used were all purchased from Merck. A full account of the synthetic procedures is given in the Supporting Information.

**Analytical instruments**: ^1^H and ^13^C NMR was performed using either a BRUKER 400 MHz or 600 MHz magnet, the spectra obtained are calibrated against residual solvent peak of CDCl_3_ (7.26/77.16 ppm), CD_2_Cl_2_ (5.32/53.84 ppm), DMSO‐*d*_6_ (2.50/39.52 ppm), Acetone‐*d*_6_ (2.05/29.84 ppm) or THF‐*d*_8_ (1.72/25.31 ppm). The IR‐spectra are obtained using a BRUKER Alpha Eco‐ATR FTIR spectrometer, the data is reported with wavenumber and intensity of the signal. Mass determination was performed by MS‐analysis in positive ionization mode on a Synapt G2‐S Q‐TOF‐instrument for Waters, the samples were ionized by an ASAP‐probe (APCI) without prior chromatographic separation. UV‐Vis spectroscopy was performed using a Hitachi U‐1900 spectrometer, and photoluminescence characterization was carried out on an Edinburgh Instruments FS5‐spectrofluorometer. CV experiments were carried out using a Versastat 3 Potentiostat, the data was acquired using the Versastudio software. The three‐electrode system consisted of a stained TiO_2_ photoanode as the working electrode, a graphite carbon counter electrode, and a Ag/AgCl reference. The supporting electrolyte was 0.1 m LiTFSI in dry acetonitrile.

**Fabrication of dye‐sensitized solar cells**: The anodes were prepared from FTO glass (NSG10, Nippon Sheet Glass), which was first cleaned in a Deconex 21‐solution (2 g/L) under sonication for 45 minutes. Next the FTO was treated with UV/O_3_ (Novascan PSD‐UV) for 15 minutes. Immersion of the glass in aqueous TiCl_4_‐solution (40 mm) at 70 °C for 2×30 minutes followed by rinsing with deionized water and ethanol was carried out before sintering the TiO_2_ for 1 h at 250 °C on hotplate to deposit a blocking layer on the FTO‐sample. Pastes of TiO_2_ were screen printed onto the FTO (120 T mesh, area 0.2826 cm^2^, Seritec Services S.A.), first two active layers (30NR‐D, Dyesol) were printed with 5 minutes heating on a hotplate at 125 °C after each layer. A scattering layer (WER2‐O, Dyesol) was ultimately printed, and the TiO_2_ was sintered on a programmable hotplate at set temperatures of 125, 250, 375, 450, and 500 °C for 5, 5, 5, 15, and 30 minutes with a ramping time of 5 minutes. This yielded electrodes consisting of a 4 μm thick active layer and 2 μm thick scattering layer measured by a Bruker DekTak XT profilometer. Before staining the electrodes were annealed at 500 °C for 30 minutes, using a hotplate.

The counter electrodes were prepared from TEC10 FTO glass supplied by Sigma Aldrich. Holes were drilled into the electrodes from the FTO‐side using a diamond drill bit, this procedure was carried out under water. The glass plates were then cleaned using Deconex 21 (aq., 2 g/L), deionized water, ethanol, and acetone, in an ultrasonic bath for 15 minutes for each. An electrocatalytic layer of poly(3,4‐ethylenedioxythiophene) was deposited on the FTO following the previously reported procedure by Ellis et al.^[48]^

The photoanodes were placed in the dye bath while still holding ∼80 °C from the annealing‐procedure and stored in a chamber at 30 °C overnight. The dye baths were prepared using a mixture of acetonitrile, *t*‐butanol, and THF (1 : 1 : 1, v/v) to make a solution of dye (0.1 mm) and any eventual co‐adsorbent CDCA or ADAA (1 mm). The staining of the reference **Y123** was done similarly, but the solvent mixture used was in this case *t*‐butanol and acetonitrile (1 : 1, v/v) and the concentration was CDCA was reduced (0.4 mm). Following 15 h of staining the electrodes were rinsed in acetonitrile for 2 minutes, then sealed to the counter electrode using Surlyn (25 μm, Solaronix) in a drybox. A 4×20 second treatment of the cell using a 50 W PTC heat element was sufficient to seal the cells. The electrolyte was vacuum backfilled into the device, the filling‐hole was sealed with Surlyn and a glass cover disk. To complete the devices, the electrodes were painted with silver conducting paint (Electrolube, SCP). The electrolyte employed was a previously reported electrolyte, consisting of [Cu^(I)^(tmby)_2_]TFSI (0.20 m), [Cu^(II)^(tmby)_2_](TFSI)_2_ (0.10 m), LiTFSI (0.10 m), and, *N*‐methylbenzimidazole (0.60 m) dissolved in dry acetonitrile.^[6]^

**Device characterization**: J‐V curves were obtained under 1 sun illumination AM 1.5G illumination provided by a Sciencetech SP300B solar simulator, calibrated with a Newport Reference Cell (91150 V), connected to a Keithley 2450 SourceMeter. A mask with an active area of 0.238 cm^2^ was used on all the J‐V measurements. IPCE measurements were carried out using a halogen lamp (Ocean Optics HL‐2000) and a monochromator (Spectral Products CM110) connected to the Keithley 2450. The devices and the reference photodiode (Thorlabs, FDS100‐CAL) were covered with a mask with a size of 0.049 cm^2^.

The electrochemical impedance properties were measured in a light‐exclusion box containing a Zahner CIMPS QE/IPCE TLS03 tunable light source. The light source was connected to a Zahner XPOT potentiostat, the devices were connected to a Zahner IM6ex potentiostat, both potentiostats were controlled by the Thales software. EIS was performed under constant illumination at wavelength 479 nm with an intensity of 12.6 mW/cm^2^. The voltage across the cells was oscillating with an amplitude of 10 mV over a 100 mHz–100 kHz frequency range. The measurements were distributed logarithmically over this range with 8 measurements per decade for frequencies higher than 66 Hz and 3 measurements per decade for frequencies lower than 66 Hz. The results above 66 Hz were also averaged over 20 measurements for every frequency while the results below 66 Hz were averaged over 2 measurements per frequency. The oscillating voltage across the DSSCs had an applied DC‐bias during the measurements which was decreased by 20 mV between measurements starting at the open circuit voltage of the cells. All DSSCs were measured 10 times each, with the DC‐bias voltage as the variable.

The resulting Nyquist plots were fitted to a transmission line model as shown by Fabregat et al.^[49]^ using the nonlinear Levenberg–Marquardt least square fitting method. From the fitted curves, the recombination resistance was extracted and plotted as a function of applied potential.

## Conflict of interest

The authors declare no conflict of interest.

1

## Supporting information

As a service to our authors and readers, this journal provides supporting information supplied by the authors. Such materials are peer reviewed and may be re‐organized for online delivery, but are not copy‐edited or typeset. Technical support issues arising from supporting information (other than missing files) should be addressed to the authors.

Supporting Information

## Data Availability

The data that support the findings of this study are available in the supplementary material of this article.
