# Supplementary material for: Adamantyl Side Chains as Anti‐Aggregating Moieties in Dyes for Dye‐Sensitized Solar Cells
Source: Chemistry. 2022 Jul 20;28(51):e202201726. doi: 10.1002/chem.202201726 (PMC9543767; doi:10.1002/chem.202201726)
Supplement: Supplementary file 1 — Supporting Information [file CHEM-28-0-s001.pdf]

Chemistry–A European Journal

Supporting Information

Adamantyl Side Chains as Anti-Aggregating Moieties in Dyes for Dye-Sensitized Solar Cells

David Moe Almenningen, Brita Susanne Haga, Henrik Erring Hansen, Audun Formo Buene, Bård Helge Hoff, and Odd Reidar Gautun*

List of contents

Absorption and emission **2**

Cyclic voltammetry **3**

Electrochemical impedance spectroscopy **4**

Experimental **5**

NMR **18**

Absorption and emission

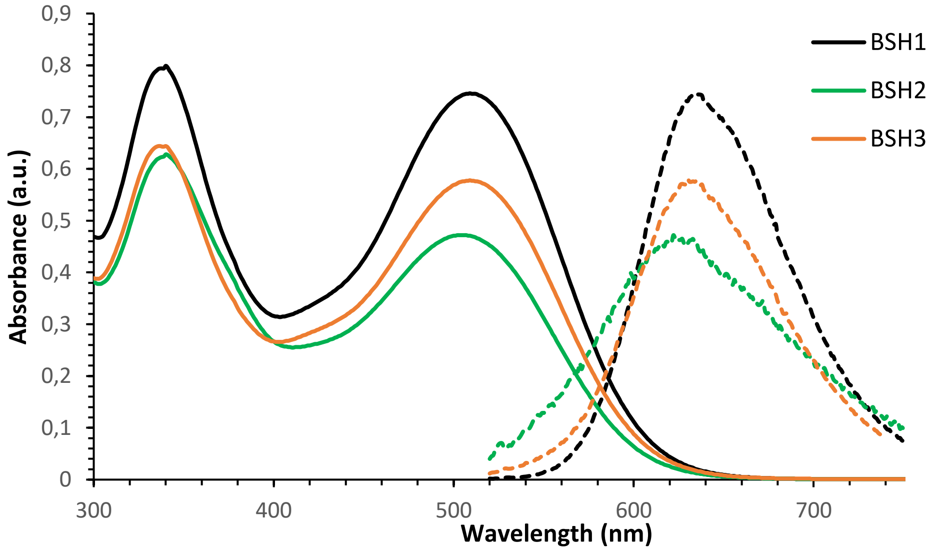

Figure S1. Normalized absorption (-) and emission (--) spectra of the dyes in DCM solution.

Cyclic voltammetry

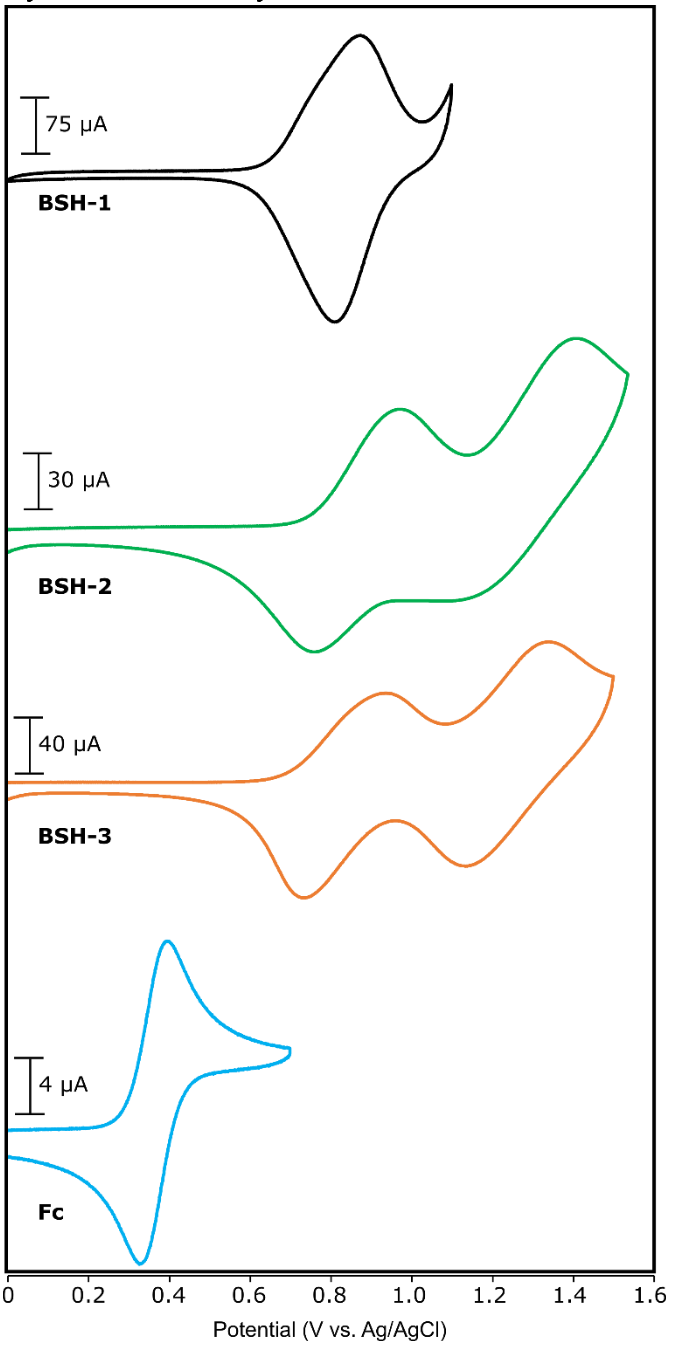

Figure S2. CVs of the dyes and ferrocene. Sensitizers measured on TiO_2 films (2.5 µm on FTO glass), carbon counter electrode, Ag/AgCl reference, 0.1 M LiTFSI supporting electrolyte. Ferrocene (Fc) was measured with a glassy carbon working electrode and used for calibration with a value of 0.624 V versus SHE.[1]

Electrochemical impedance spectroscopy

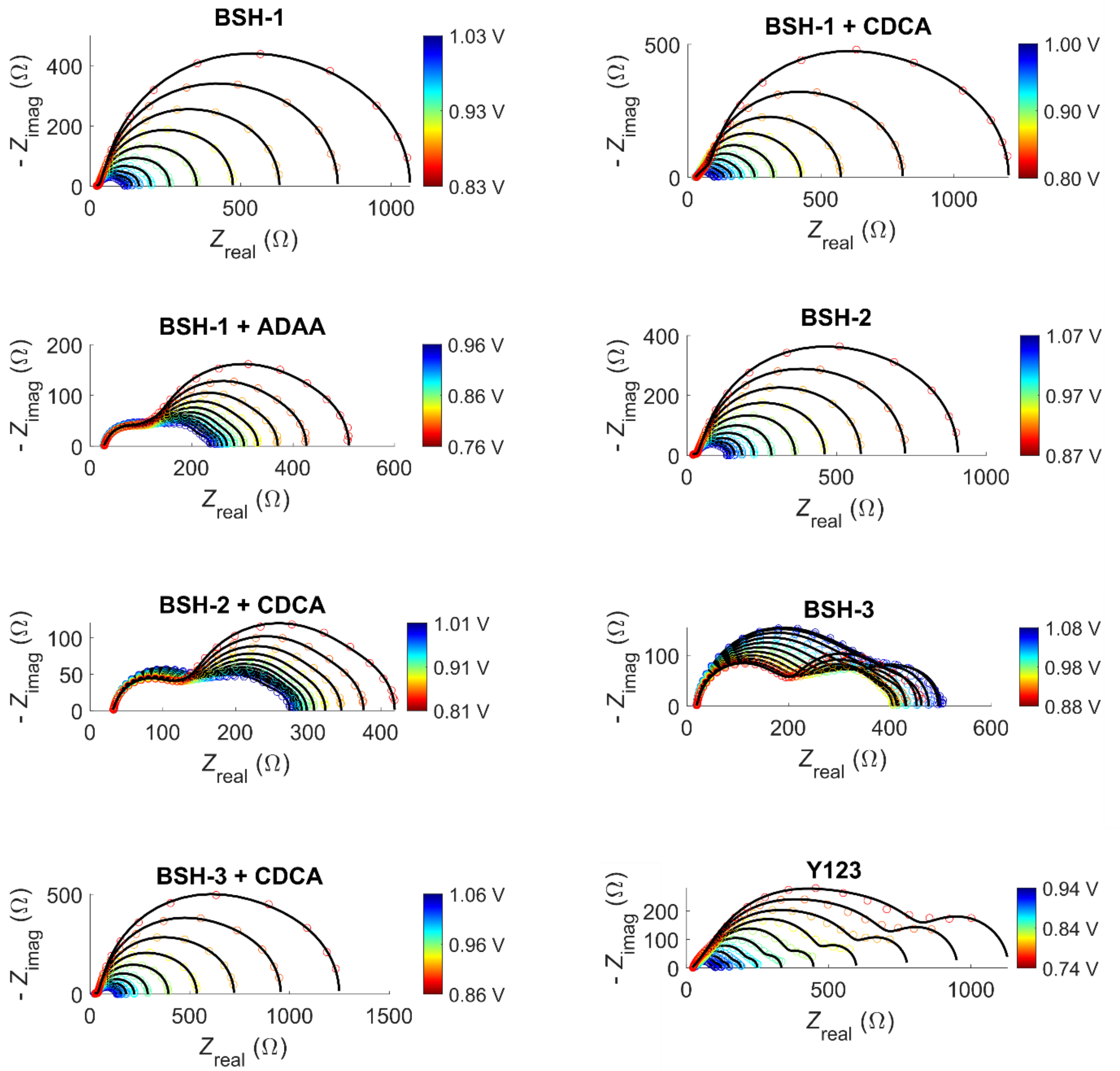

Figure S3. Complex plane diagrams obtained from electrochemical impedance spectroscopy under monochromatic light of 479 nm.

Experimental

Materials and reagents

All reactions were carried out under nitrogen atmosphere, and all synthesis reagents were acquired from Merck.

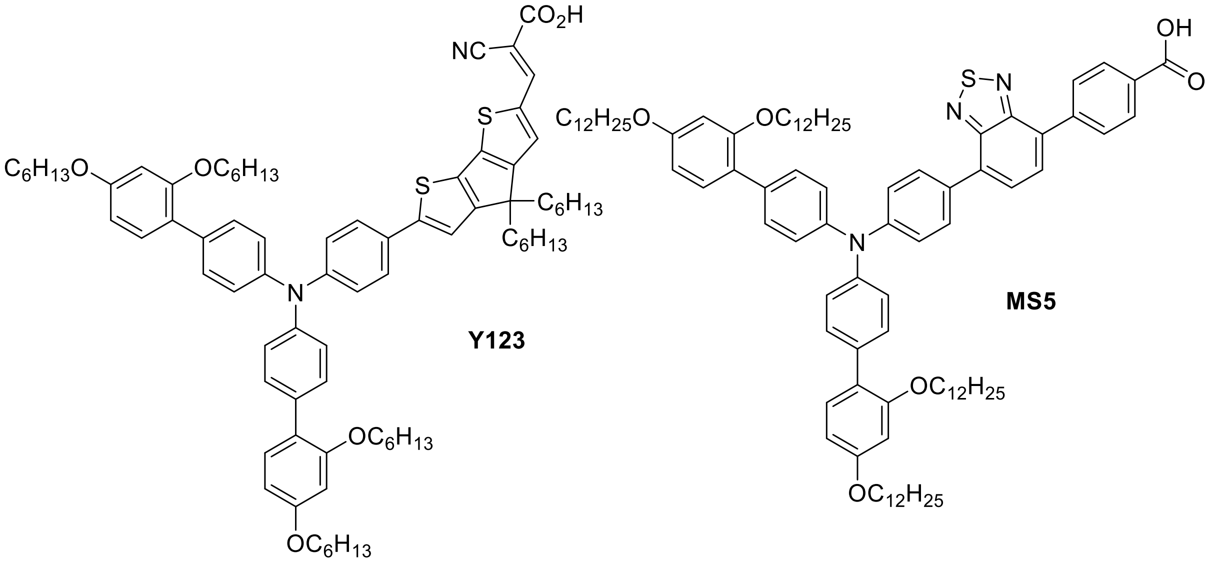

Figure S4. Structure of the benchmark dye **Y123**[2] and the dye **MS5**[3] which was a source of inspiration for the **BSH-3** dye.

Analytical instruments

NMR spectroscopy (^{1}H and ^{13}C) was recorded on 400 and 600 MHz Bruker instruments, and all chemical shifts are reported relative to the respective solvent signals. Mass determination was performed on a Waters "Synapt G2-S" QTOF instrument in positive and negative modes. UV/Vis spectra were recorded on a Hitachi U-1900 instrument using quarts cuvettes for the solution samples, while fluorescence spectroscopy was recorded on a Cary Eclipse Fluorescence Spectrophotometer. Infrared spectroscopy was recorded on an FTIR Thermo Nicolet Nexus FT-IR spectrophotometer.

Synthesis of methyl 2-(adamantan-1-yl)acetate (1)

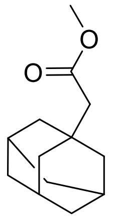

1-Adamantaneacetic acid (6.00 g, 30.9 mmol) was dissolved in methanol (110 mL), before dropwise addition of H_2SO_4 (95%, 1.6 mL). The resulting mixture was stirred at r.t. overnight. Following this, the solvent was removed under reduce pressure. The residue was dissolved in ethyl acetate (120 mL) before being washed with an aqueous solution of $NaHCO_3$ (0.5 M, 120 mL) The aqueous phase was extracted using ethyl acetate (3 × 100 mL), and the combined organic phases dried over Na_2SO_4. The organic phase was then filtered, and the solvents were removed *in vacuo*. The crude product was purified by silica gel column chromatography (dichloromethane, $R_f = 0.8$). The product **1** was isolated as a clear oil (5.10 g, 24.5 mmol, 79%). ^{1}H NMR (400 MHz, $CDCl_3$) δ: 3.63 (s, 3H), 2.06 (s, 2H), 1.98-1.90 (m, 3H), 1.72-1.54 (m, 12H); ^{13}C NMR (150 MHz, $CDCl_3$) δ: 172.4, 51.1, 48.9, 42.5 (3C), 36.8 (3C), 32.8, 28.7 (3C);

IR (neat, cm^{-1}) ν: 2899 (s), 2847 (m), 1734 (s), 1448 (m), 1325 (m), 1137 (s), 1018 (s); HRMS (ASAP+, *m/z*): found 209.1544 (calcd. $C_{13}H_{21}O_2$ 209.1542, $[M+H]^+$).

Synthesis of 1-adamantaneethanol (2)

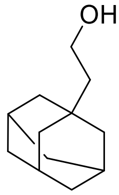

A suspension was of $LiAlH_4$ (1.34 g, 35.3 mmol) in anhydrous THF (50 mL) was kept at 0 ºC, to which a solution of **1** (5.10 g, 24.5 mmol) in anhydrous THF (15 mL) was added slowly. The resulting mixture was stirred overnight while being warmed to room temperature. The reaction was stopped following a modified Fieser work-up procedure where water (3.2 mL), then aqueous NaOH (4 M, 3.2 mL) was added to form a white granulate. Water (8 mL) was then added and the reaction mixture was stirred for 10 minutes, before addition of anhydrous Na2SO4 and 10 minutes stirring before removal of solids by filtration. The solvents were removed *in vacuo*, yielding the product **2** as a white solid (3.48 g, 19.3 mmol, 79%). ^{1}H NMR (400 MHz, $CDCl_3$) δ: 3.69 (t, *J* = 7.5 Hz, 2H), 1.96-1.89 (m, 3H), 1.73-1.58 (m, 6H) 1.54-1.49 (m, 6H), 1.37 (t, *J* = 7.5 Hz, 2H) (-O<u>H</u> proton missing). The NMR spectrum is in accordance with previously reported data.[4]

Synthesis of 1-(2-bromoethyl)adamantane (3)

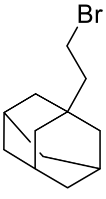

Compound **2** (2.00 g, 11.1 mmol) was added aqueous HBr (47%, 30 mL) and refluxed at 100 ºC overnight. After being cooled to room temperature the reaction mixture was diluted with water (50 mL). The aqueous phase was extracted using dichloromethane (3 × 80 mL), and the combined organic phases were dried over Na_2SO_4. The organic phase was then filtered, and the solvents were removed *in vacuo*, yielding **3** as a white solid (2.54 g, 10.5 mmol, 94%). ^{1}H NMR (400 MHz, $CDCl_3$) δ: 3.44-3.36 (m, 2H), 1.99-1.91 (m, 3H), 1.75-1.57 (m, 8H), 1.53-1.47 (m, 6H). The NMR spectrum is in accordance with previously reported data.[5]

Synthesis of 3-((2-(adamantan-1-yl)ethoxy)methyl)-2-bromothiophene (4)

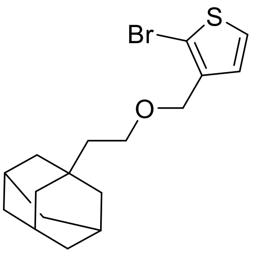

Compound **3** (3.48 g, 19.3 mmol), NaH (460 mg, 19.3 mmol), NaI (580 mg, 3.9 mmol) and anhydrous THF (28 mL) were placed in a flask under nitrogen atmosphere and stirred at 0 °C. A solution of 2-bromo-3-bromomethylthiophene (4.9 mL, 19.3 mmol) in anhydrous THF (30 mL) was added dropwise before the reaction was heated to 50 °C and stirred overnight. The reaction mixture was cooled to room temperature and was quenched by addition of water (60 mL). The aqueous phase was extracted with ethyl acetate (3 × 60 mL) and dried over

anhydrous Na_2SO_4 before the solvents were removed in vacuo. The crude product was purified by silica gel column chromatography (*n*-pentane, $R_f = 0.58$) to obtain **4** as a yellow oil (5.16 g, 14.6 mmol, 75%). ^{1}H NMR (400 MHz, $CDCl_3$) δ: 7.23 (d, *J* = 5.8 Hz, 1H), 6.98 (d, *J* = 5.8 Hz, 1H), 4.42 (s, 2H), 3.52 (t, *J* = 8.3 Hz, 2H), 1.97-1.90 (m, 3H), 1.73-1.59 (m, 6H), 1.53-1.48 (m, 6H), 1.41 (t, *J* = 8.3 Hz, 2H); ^{13}C NMR (100 MHz, $CDCl_3$) δ: 138.8, 128.4, 126.0, 110.9, 67.0, 66.6, 43.7, 42.9 (3C), 37.3 (3C), 31.9, 28.8 (3C); IR (neat, cm^{-1}) ν: 2896 (s), 2844 (s), 1449 (m), 1103 (s), 686 (m).

Synthesis of 3'-((2-(adamantan-1-yl)ethoxy)methyl)-[2,2'-bithiophene]-5-carbaldehyde (5)

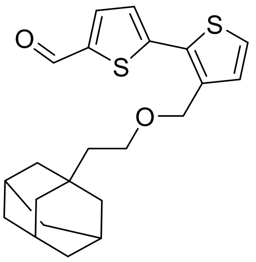

Compound **4** (300 mg, 0.84 mmol) and 5-formyl-2-thiopheneboronic acid (190 mg, 1.27 mmol), K_3PO_4 (1.54 g, 11.1 mmol), and the precatalyst XPhos Pd G3 (22 mg, 26 µmol) were added to a Schlenk tube and N_2-atmosphere established. 1,4-Dioxane (3 mL) and water (3 mL) were degassed and added under nitrogen, the reaction mixture was stirred at 80 °C for 2 hours. After cooling to room temperature, water (10 mL) was added and the aqueous phase was extracted with ethyl acetate (3 x 10 mL). The combined organic phases were dried with brine (10 mL) and over anhydrous Na_2SO_4, filtered and the solvents were removed *in vacuo.* The residue was purified by silica gel column chromatography (ethyl acetate/*n*-pentane, 1:10, R_f = 0.31). The product **5** was isolated as an orange oil (170 mg, 0.45 mmol, 54%). ^{1}H NMR (400 MHz, $CDCl_3$) δ: 9.84 (s, 1H), 7.67 (d, *J* = 3.4 Hz, 1H), 7.27 (d , *J* = 5.4 Hz, 1H), 7.25 (d, *J* = 3.4 Hz, 1H), 7.11 (d, *J* = 5.4 Hz, 1H), 4.52 (s, 2H), 3.54 (t, *J* = 7.6 Hz, 2H) 1.93-1.84 (m, 3H), 1.69-1.53 (m, 6H), 1.49-1.44 (m, 6H), 1.40 (t, *J* = 7.6 Hz, 2H); ^{13}C NMR (100 MHz, $CDCl_3$) δ: 182.8, 145.7, 143.2, 138.1, 137.0, 132.6, 130.8, 127.0, 126.1, 66.82, 66.75, 43.7, 42.8 (3C), 37.2 (3C), 31.9, 28.8 (3C); IR (neat, cm^{-1}) ν: 2899 (s), 2845 (s), 1666 (s), 1449 (s), 1226 (w); HRMS (ASAP+, *m/z*): found 387.1452 (calcd. $C_{22}H_{27}O_2S_2$ 387.1452, $[M+H]^+$).

Synthesis of 3'-((2-(adamantan-1-yl)ethoxy)methyl)-5'-bromo-[2,2'-bithiophene]-5-carbaldehyde (6)

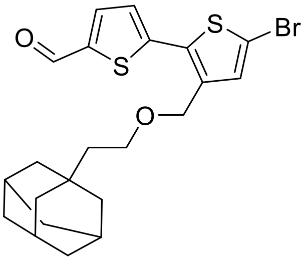

Compound **5** (170 mg, 0.48 mmol) and NBS (110 mg, 0.58 mmol) were added to a round-bottom flask under dark conditions. Degassed dichloromethane (8 mL) was degassed and added under nitrogen at 0 °C. The reaction mixture was then allowed to warm to room temperature while stirring for 15 hours. Water (20 mL) was added and the aqueous phase was extracted

using dichloromethane (3 × 15 mL). The combined organic phases were dried over anhydrous Na_2SO_4, filtered and the solvents were removed *in vacuo*. The crude product was purified by silica gel column chromatography (ethyl acetate/*n*-pentane 1:10, R_f = 0.34). Compound **6** was isolated as a red oil (140 mg, 0.31 mmol, 66%). ^{1}H NMR (400 MHz, $CDCl_3$) δ: 9.89 (s, 1H), 7.70 (d, *J* = 4.0 Hz, 1H), 7.21 (d , *J* = 4.0 Hz, 1H), 7.13 (s, 1H), 4.49 (s, 2H), 3.56 (t, *J* = 7.4 Hz, 2H) 1.96-1.91 (m, 3H), 1.73-1.59 (m, 6H), 1.52-1.51 (m, 6H), 1.43 (t, *J* = 7.4 Hz, 2H); ^{13}C NMR (100 MHz, $CDCl_3$) δ: 182.7, 144.0, 143.5, 138.7, 136.8, 133.6, 133.3, 127.1, 113.4, 67.0, 66.5, 43.7, 42.8 (3C), 37.2 (3C), 31.9, 28.8 (3C); IR (neat, cm^{-1}) ν: 2899 (s), 2845 (s), 1668 (s), 1450 (s), 1220 (w); HRMS (ASAP+, *m/z*): found 465.0558 (calcd. $C_{22}H_{26}BrO_2S_2$ 465.0557, $[M+H]^+$).

Synthesis of 1-bromo-2,4-bis(hexyloxy)benzene (7)

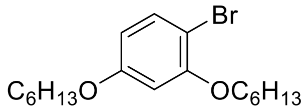

4-Bromoresorcinol (2.99 g, 15.8 mmol) and KOH (7.84 g, 47.5 mmol) were dissolved in DMSO (30 mL), before 1-bromohexane (6.7 mL, 47.5 mmol) was added. The resulting mixture was stirred at r.t. overnight. Following this, the mixture was quenched in water (20 mL). The aqueous phase was extracted using *n*-pentane (4 × 30 mL), the combined organic phases were washed with water (3 x 40 mL) before being dried with brine (50 mL) and over Na_2SO_4. The organic phase was then filtered, and the solvents were removed *in vacuo*. The crude product was purified by silica gel column chromatography (dichloromethane/*n*-pentane, 1:5, R_f = 0.27). The product **7** was isolated as a clear oil (4.65 g, 13.0 mmol, 82%). ^{1}H NMR (400 MHz, DMSO-*d*$_6$) δ: 7.39 (d, *J* = 8.7 Hz, 1H), 6.62 (d, *J* = 2.6 Hz, 1H), 6.46 (dd, *J* = 8.7, 2.7 Hz, 1H), 4.01 (t, *J* = 6.4 Hz, 2H), 3.94 (t, *J* = 6.5 Hz, 2H), 1.74-1.64 (m, 4H), 1.47-1.35 (m, 4H), 1.34-1.26 (m, 8H), 0.89-0.85 (m, 6H). The NMR spectrum is in accordance with previously reported data.[6]

Synthesis of 1,1'-(((4-bromo-1,3-phenylene)bis(oxy))bis(ethane-2,1-diyl))bis(adamantane) (9)

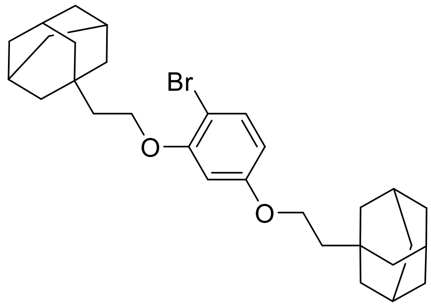

4-Bromoresorcinol (0.52 g, 2.7 mmol), compound **3** (2.00 g, 8.2 mmol) and KOH (0.46 g, 8.2 mmol) were dissolved in DMSO (50 mL). The resulting mixture was stirred at r.t. overnight. Following this, the mixture was quenched in water (100 mL). The aqueous phase was extracted using *n*-pentane (4 × 50 mL), the combined organic phases were washed with water (3 x 40 mL) before being dried with brine (40 mL) and over Na_2SO_4. The organic phase was then filtered, and the solvents were removed *in vacuo*. The crude product was purified by silica gel column chromatography (dichloromethane/*n*-pentane, 1:5, R_f = 0.24). The product **9** was isolated as a white solid (0.69 g, 1.3 mmol, 49%) mp 85-88 °C. ^{1}H NMR (600 MHz, $CDCl_3$) δ: 7.37 (d, *J* = 8.7 Hz, 1H), 6.45 (d, *J* = 2.7 Hz, 1H), 6.36 (dd, *J* = 8.7, 2.7 Hz, 1H), 4.05 (t, *J* = 7.2

Hz, 2H), 3.99 (t, *J* = 7.2 Hz, 2H), 1.98-1.95 (m, 6H), 1.74-1.69 (m, 6H), 1.68-1.63 (m, 8H), 1.62-1.55 (m, 14H); ^{13}C NMR (150 MHz, $CDCl_3$) δ: 159.7, 156.3, 133.2, 106.4, 102.8, 101.4, 65.2, 64.5, 43.1, 42.84 (3C), 42.83 (3C), 37.21 (3C), 37.19 (3C), 32.0, 28.81 (3C), 28.78 (3C); IR (neat, cm^{-1}) ν: 2945 (s), 2845 (m), 1597 (w), 1305 (w), 1019 (w); HRMS (ASAP+, *m/z*): found 513.2361 (calcd. $C_{30}H_{42}BrO_2$ 513.2368, $[M+H]^+$).

Synthesis of *N*-(2',4'-bis(hexyloxy)-[1,1'-biphenyl]-4-yl)-2',4'-bis(hexyloxy)-*N*-phenyl-[1,1'-biphenyl]-4-amine (11)

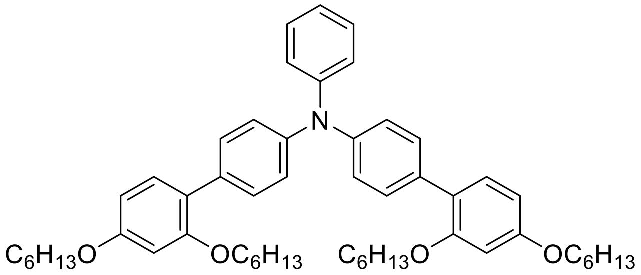

Compound **7** (1.99 g, 12.9 mmol) was added along with $PdCl_2(CH_3CN)_2$ (44 mg, 170 µmol), SPhos (138 mg, 336 µmol). The compounds were dissolved in 1,4-dioxane (17 mL), then 4,4,5,5-tetramethyl-1,3,2-dioxaborolane (1.6 mL, 11.8 mmol) and NEt_3 (2.3 mL, 17.6 mmol) were added. The reaction mixture was heated to 80 °C and left stirring for two hours before the reaction mixture was cooled to room temperature. The contents of the reaction were filtered through a Celite plug using EtOAc as eluent, the solvents were then removed in vacuo. The crude boronate ester **8** was a brown viscous oil that was used without further purification.

4,4′-Dibromotriphenylamine (471 mg, 1.2 mmol), K_2CO_3 (1.54 g, 11.1 mmol), $Pd(OAc)_2$ (19 mg, 84 µmol), SPhos (69 mg, 168 µmol) along with 1.51 g of the crude boronate **8** were added. 1,4-Dioxane (5 mL) and water (5 mL) were degassed and added under nitrogen, the reaction mixture was stirred at 80 °C for 24 hours. After cooling to room temperature, water (50 mL) was added and the aqueous phase was extracted with ethyl acetate (3 x 50 mL). The combined organic phases were dried with brine (50 mL) and over anhydrous Na_2SO_4, filtered and the solvents were removed *in vacuo.* The residue was purified by silica gel column chromatography (dichloromethane/*n*-pentane, 1:1, R_f = 0.40). The product **11** was isolated as a yellow oil (810 mg, 1.00 mmol, 87%). ^{1}H NMR (400 MHz, $CDCl_3$) δ: 7.45-7.40 (m, 4H), 7.28-7.22 (m, 4H), 7.20-7.16 (m, 2H), 7.15-7.10 (m, 4H), 7.00 (t, *J* = 6.8 Hz, 1H), 6.56-6.51 (m, 4H), 4.00-3.92 (m, 8H), 1.84-1.69 (m, 8H), 1.52-1.44 (m, 4H), 1.44-1.23 (m, 20H), 0.91 (t, *J* = 7.1 Hz, 6H), 0.86 (t, *J* = 6.9 Hz, 6H). The NMR spectrum is in accordance with previously reported data.[6]

Synthesis of 2',4'-bis(2-(adamantan-1-yl)ethoxy)-*N*-(2',4'-bis(2-(adamantan-1-yl)ethoxy)-[1,1'-biphenyl]-4-yl)-*N*-phenyl-[1,1'-biphenyl]-4-amine (12)

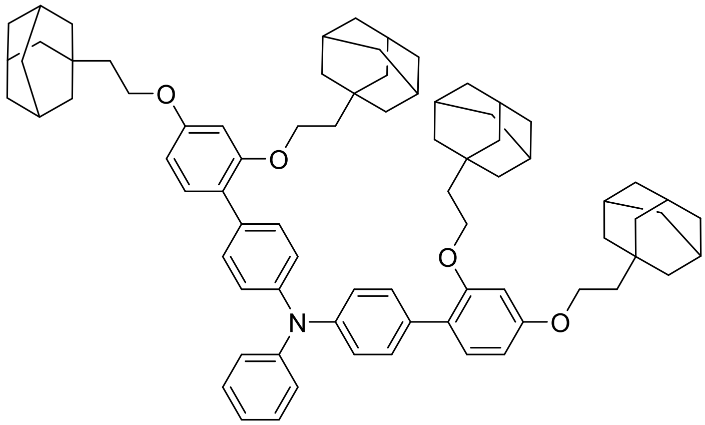

Compound **9** (686 mg, 1.3 mmol) was added along with $PdCl_2(CH_3CN)_2$ (19 mg, 72 µmol), SPhos (59 mg, 144 µmol). The compounds were dissolved in 1,4-dioxane (6 mL), then 4,4,5,5-tetramethyl-1,3,2-dioxaborolane (0.42 mL, 2.9 mmol) and NEt_3 (0.6 mL, 4.3 mmol) were added. The reaction mixture was heated to 80 °C and left stirring for two hours before the reaction mixture was cooled to room temperature. The contents of the reaction were filtered through a Celite plug using EtOAc as eluent, the solvents were then removed in vacuo. The crude boronate **10** was a brown viscous oil and was used without further purification.

4,4′-Dibromotriphenylamine (250 mg, 0.62 mmol), K_2CO_3 (750 mg, 5.4 mmol), $Pd(OAc)_2$ (9 mg, 40 µmol), SPhos (69 mg, 80 µmol) along with 0.74 g of the crude boronate **10** were added. 1,4-Dioxane (3 mL) and water (2 mL) were degassed and added under nitrogen, the reaction mixture was stirred at 80 °C for 24 hours. After cooling to room temperature, water (50 mL) was added and the aqueous phase was extracted with dichloromethane (3 x 30 mL). The combined organic phases were dried over anhydrous Na_2SO_4, filtered and the solvents were removed *in vacuo.* The residue was purified by silica gel column chromatography (dichloromethane/*n*-pentane, 1:4, R_f = 0.14). The product **12** was isolated as a white solid (390 mg, 0.35 mmol, 56%) mp 120-124 °C. ^{1}H NMR (600 MHz, $CDCl_3$) δ: 7.41 (app. d, *J* = 8.6 Hz, 4H), 7.27-7.24 (m, 4H), 7.17 (d, *J* = 8.3 Hz, 2H), 7.12 (app. d, *J* = 8.6 Hz, 4H), 7.00 (t, *J* = 8.3 Hz, 1H), 6.55-6.52 (m, 4H), 4.06 (t, *J* = 7.0 Hz, 4H), 4.01 (t, *J* = 7.0 Hz, 4H), 2.00-1.97 (m, 6H), 1.94-1.92 (m, 6H), 1.75-1.64 (m, 24H), 1.62-1.59 (m, 16H), 1.58-1.54 (m, 4H), 1.53-1.50 (m, 12H); ^{13}C NMR (150 MHz, $CDCl_3$) δ: 159.6 (2C), 157.2 (2C), 148.2, 146.2 (2C), 133.0 (2C), 131.0 (2C), 130.4 (4C), 129.3 (2C), 124.3 (2C), 123.7 (4C), 123.4 (2C), 122.5, 105.3 (2C), 100.5 (2C), 64.7 (2C), 64.2 (2C), 43.2 (2C), 43.0 (2C), 42.9 (6C), 42.8 (6C), 37.2 (12C), 32.0 (2C), 31.9 (2C), 28.81 (6C), 28.80 (6C); IR (neat, cm^{-1}) ν: 2897 (s), 1845 (m), 1606 (m), 1492 (m), 1175 (m); HRMS (ASAP+, *m/z*): found 1109.7261 (calcd. $C_{78}H_{95}NO_4$ 1109.7240, $[M]^+$).

Synthesis of *N*-(2',4'-bis(hexyloxy)-[1,1'-biphenyl]-4-yl)-*N*-(4-bromophenyl)-2',4'-bis(hexyloxy)-[1,1'-biphenyl]-4-amine (13)

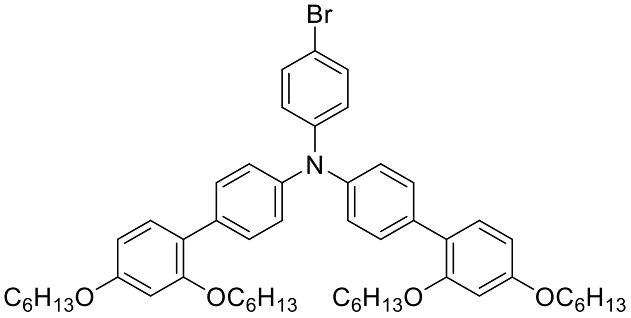

Compound **11** (0.81 g, 1.01 mmol) and NBS (0.18 g, 1.01 mmol) were added to a round-bottom flask under dark conditions. Dichloromethane (20 mL) was degassed and added under nitrogen at 0 °C. The reaction mixture was then allowed to warm to room temperature while stirring for 15 hours. Water (40 mL) was added and the aqueous phase was extracted using dichloromethane (3 × 30 mL). The combined organic phases were dried over anhydrous Na_2SO_4, filtered and the solvents were removed *in vacuo*. The crude product was purified by silica gel column chromatography (dichloromethane, R_f = 0.8). Compound **13** was isolated as a clear oil (0.87 g, 0.99 mmol, 99%). ^{1}H NMR (400 MHz, $CDCl_3$) δ: 7.46-7.41 (m, 4H), 7.35-7.30 (m, 2H), 7.27-7.23 (m, 2H), 7.14-7.08 (m, 4H), 7.06-7.02 (m, 2H), 6.56-6.51 (m, 4H), 4.00-3.92 (m, 8H), 1.84-1.70 (m, 8H), 1.52-1.44 (m, 4H), 1.44-1.25 (m, 20H), 0.91 (t, J = 6.5 Hz, 6H), 0.87 (t, J = 6.5 Hz, 6H). The NMR spectrum is in accordance with previously reported data.[6]

Synthesis of *N*-(2',4'-bis(hexyloxy)-[1,1'-biphenyl]-4-yl)-*N*-(4-bromophenyl)-2',4'-bis(hexyloxy)-[1,1'-biphenyl]-4-amine (14)

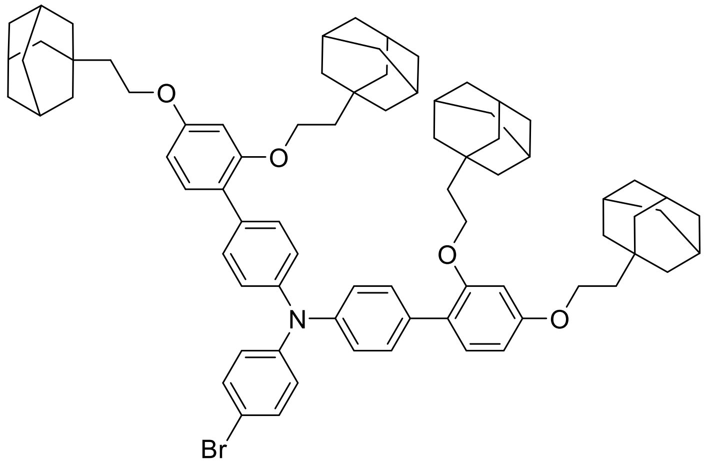

Compound **12** (370 mg, 0.34 mmol) and NBS (64 mg, 0.36 mmol) were added to a round-bottom flask under dark conditions. Dichloromethane (8 mL) was degassed and added under nitrogen at 0 °C. The reaction mixture was then allowed to warm to room temperature while stirring for 15 hours. Water (20 mL) was added and the aqueous phase was extracted using dichloromethane (3 × 15 mL). The combined organic phases were dried over anhydrous Na_2SO_4, filtered and the solvents were removed *in vacuo*. The crude product was purified by silica gel column chromatography (dichloromethane, R_f = 0.84). Compound **14** was isolated as

a white solid (0.38 g, 0.32 mmol, 95%) mp 139-141 °C. ^{1}H NMR (600 MHz, $CDCl_3$) δ: 7.42 (d, J = 9.5 Hz, 4H), 7.32 (d, J = 9.1 Hz, 2H), 7.24 (d, J = 8.1 Hz, 2H), 7.10 (d, J = 9.5 Hz, 4H), 7.03 (d, J = 9.1 Hz, 2H), 6.55-6.52 (m, 4H), 4.06 (t, J = 7.4 Hz, 4H), 4.02 (t, J = 7.2 Hz, 4H), 2.00-1.96 (m, 6H), 1.95-1.90 (m, 6H), 1.76-1.64 (m, 20H), 1.64-1.58 (m, 20H), 1.56 (t, J = 7.3 Hz, 4H), 1.52-1.49 (m, 12H); ^{13}C NMR (150 MHz, $CDCl_3$) δ: 159.6 (2C), 157.2 (2C), 147.4, 145.6 (2C), 133.6 (2C), 132.2 (2C), 131.0 (2C), 130.5 (4C), 125.2 (2C), 123.9 (4C), 123.1 (2C), 114.5, 105.3 (2C), 100.5 (2C), 64.7 (2C), 64.2 (2C), 43.2 (2C), 43.0 (2C), 42.9 (6C), 42.8 (6C), 37.22 (6C), 37.21 (6C), 32.0 (2C), 31.9 (2C), 28.81 (6C), 28.80 (6C); IR (neat, cm^{-1}) ν: 2896 (s), 2844 (m), 1604 (m), 1580 (m), 1468 (m), 1264 (s), 1174 (m), 1019 (m), 835 (m), 737 (s); HRMS (ASAP+, *m/z*): found 1187.6365 (calcd. $C_{78}H_{94}NO4Br$ 1187.6366, $[M]^{+}$).

Synthesis of 5'-(4-(bis(2',4'-bis(hexyloxy)-[1,1'-biphenyl]-4-yl)amino)phenyl)-[2,2'-bithiophene]-5-carbaldehyde (15)

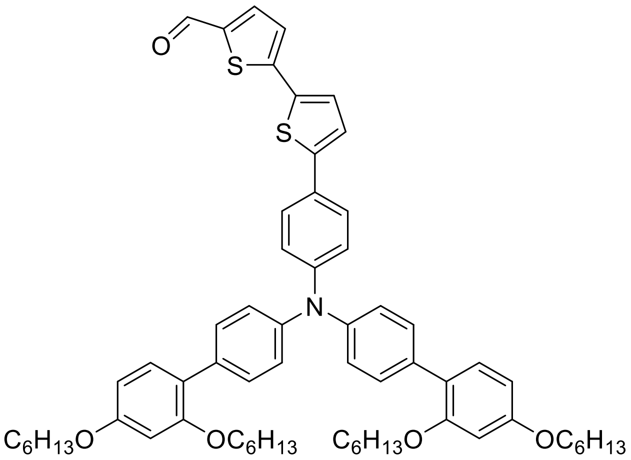

Compound 1**3** (270 mg, 0.31 mmol) was added along with $PdCl_2(CH_3CN)_2$ (2.4 mg, 9 µmol), SPhos (7.5 mg, 18 µmol). The compounds were dissolved in 1,4-dioxane (1 mL), then 4,4,5,5-tetramethyl-1,3,2-dioxaborolane (0.1 mL, 0.69 mmol) and NEt_3 (0.15 mL, 1.08 mmol) were added. The reaction mixture was heated to 110 °C and left stirring for one hour before the reaction mixture was cooled to room temperature. The contents of the reaction were filtered through a Celite plug using EtOAc as eluent, the solvents were then removed in vacuo. The crude boronic ester was a brown viscous oil that was used without further purification.

5′-Bromo-2,2′-bithiophene-5-carboxaldehyde (76 mg, 0.3 mmol), K_2CO_3 (254 mg, 1.8 mmol), $Pd(OAc)_2$ (3.5 mg, 15 µmol), SPhos (12.6 mg, 30 µmol) along with 0.28 g of the crude boronate product were added. 1,4-Dioxane (5 mL) and water (5 mL) were degassed and added under nitrogen, the reaction mixture was stirred at 80 °C for 18 hours. After cooling to room temperature, water (50 mL) was added, and the aqueous phase was extracted with dichloromethane (3 x 40 mL). The combined organic phases were dried over anhydrous Na_2SO_4, filtered and the solvents were removed *in vacuo.* The residue was purified by silica gel column chromatography (dichloromethane/*n*-pentane, 1:4, R_f = 0.48). The product **15** was isolated as an orange wax (130 mg, 0.13 mmol, 43%). ^{1}H NMR (600 MHz, $CDCl_3$) δ: 9.86 (s, 1H), 7.67 (d, J = 4.0 Hz, 1H), 7.50-7.45 (m, 6H), 7.33 (d, J = 4.0 Hz, 1H), 7.27 (app. d, J = 8.9 Hz, 2H), 7.24 (d, J = 3.7 Hz, 1H) 7.20-7.15 (m, 7H), 6.57-6.53 (m, 4H), 4.02-3.95 (m, 8H), 1.81 (q, J = 8.2 Hz, 4H), 1.76 (q, J = 8.2 Hz, 4H), 1.52-1.45 (m, 4H), 1.44-1.39 (m, 4H), 1.39-

1.34 (m, 8H), 1.34-1.28 (m, 8H), 0.93 (t, J = 7.0 Hz, 6H), 0.88 (t, J = 7.0 Hz, 6H); ^{13}C NMR (150 MHz, $CDCl_3$) δ: 182.5, 159.8 (2C), 157.1 (2C), 148.4, 147.7, 146.7, 145.5 (2C), 141.4, 137.6, 134.0, 133.8 (2C), 131.0 (2C), 130.5 (4C), 127.4, 126.9, 126.7 (2C), 124.3 (4C), 123.8, 123.20, 123.17 (2C), 123.0 (2C), 105.5 (2C), 100.6 (2C), 68.6 (2C), 68.3 (2C), 31.8 (2C), 31.6 (2C), 29.5 (2C), 29.2 (2C), 25.9 (2C), 22.8 (2C), 22.7 (2C), 14.2 (4C); IR (neat, cm^{-1}) ν: 2928 (m), 2857 (m), 1665 (m), 1453 (s), 1288 (m), 1181 (m), 1048 (m), 832 (m), 795 (m); HRMS (ASAP+, *m/z*): found 989.5075 (calcd. $C_{63}H_{75}NO_5S_2$ 989.5087, $[M]^+$). The NMR spectra are in accordance with previously reported data.[7]

Synthesis of 3'-((2-(adamantan-1-yl)ethoxy)methyl)-5'-(4-(bis(2',4'-bis(hexyloxy)-[1,1'-biphenyl]-4-yl)amino)phenyl)-[2,2'-bithiophene]-5-carbaldehyde (16)

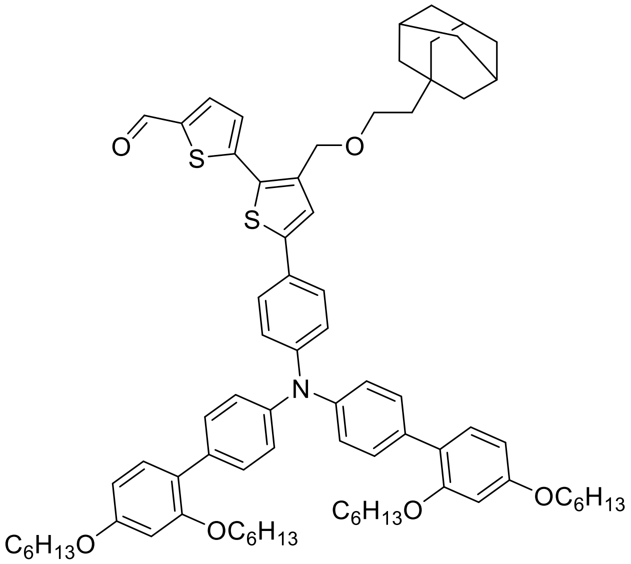

Compound 13 (270 mg, 0.31 mmol) was added along with $PdCl_2(CH_3CN)_2$ (2.4 mg, 9 µmol), SPhos (7.5 mg, 18 µmol). The compounds were dissolved in 1,4-dioxane (1 mL), then 4,4,5,5-tetramethyl-1,3,2-dioxaborolane (0.1 mL, 0.69 mmol) and NEt_3 (0.15 mL, 1.08 mmol) was added. The reaction mixture was heated to 110 °C and left stirring for one hour before the reaction mixture was cooled to room temperature. The contents of the reaction were filtered through a Celite plug using EtOAc as eluent, the solvents were then removed in vacuo. The crude boronic ester was a brown viscous oil that was used without further purification.

Compound **6** (129 mg, 0.28 mmol), K_2CO_3 (254 mg, 1.8 mmol), $Pd(OAc)_2$ (3.5 mg, 15 µmol), SPhos (12.6 mg, 30 µmol) along with 0.28 g of the crude boronate product were added. 1,4-Dioxane (5 mL) and water (5 mL) were degassed and added under nitrogen, the reaction mixture was stirred at 80 °C for 18 hours. After cooling to room temperature, water (50 mL) was added, and the aqueous phase was extracted with dichloromethane (3 x 40 mL). The combined organic phases were dried over anhydrous Na_2SO_4, filtered and the solvents were removed *in vacuo.* The residue was purified by silica gel column chromatography (dichloromethane, R_f = 0.48). The product **16** was isolated as a yellow-orange wax (170 mg, 0.15 mmol, 48%). ^{1}H NMR (600 MHz, CD_2Cl_2) δ: 9.87 (s, 1H), 7.73 (d, J = 3.8 Hz, 1H), 7.52 (d, J = 8.9 Hz, 2H), 7.48 (app. d, J = 8.3 Hz, 4H) 7.33 (d, J = 3.8 Hz, 1H), 7.32 (s, 1H), 7.25 (d, J = 8.1 Hz, 2H), 7.19-7.14 (m, 6H), 6.57-6.53 (m, 4H), 4.57 (s, 2H), 4.02-3.95 (m, 8H), 3.62 (t, J = 7.4 Hz, 2H), 1.96-1.91 (m, 3H), 1.83-1.74 (m, 8H), 1.73-1.62 (m, 6H), 1.55 (d, J = 2.5 Hz, 6H) 1.52-1.40 (m, 10H), 1.39-

1.35 (m, 8H), 1.34-1.29 (m, 8H), 0.93 (t, J = 7.0 Hz, 6H), 0.8 (t, J = 7.0 Hz, 6H); ^{13}C NMR (150 MHz, CD_2Cl_2) δ: 182.9, 160.2 (2C), 157.4 (2C), 148.6, 145.81 (2C), 145.78, 144.8, 143.1, 139.8, 137.4, 134.2 (2C), 131.2 (2C), 130.8 (4C), 130.73, 127.1, 126.9 (2C), 126.8, 125.9, 124.6 (4C), 123.4 (2C), 123.0 (2C), 105.9 (2C), 100.6 (2C), 68.8 (2C), 68.6 (2C), 67.3, 67.1, 44.1, 43.1 (3C), 37.5 (3C), 32.2, 32.1 (2C), 31.9 (2C), 29.7 (2C), 29.5 (2C), 29.3 (3C), 26.21 (2C), 26.16 (2C), 23.1 (2C), 23.0 (2C), 14.3 (2C), 14.2 (2C); IR (neat, cm^{-1}) ν: 2906 (m), 2848 (w), 1665 (m), 1602 (s), 1493 (s), 1291 (m), 1182 (m), 1055 (m); HRMS (ASAP+, *m/z*): found 1181.6589 (calcd. $C_{76}H_{95}NO_6S_2$ 1181.6601, $[M]^+$).

Synthesis of 5'-(4-(bis(2',4'-bis(2-(adamantan-1-yl)ethoxy)-[1,1'-biphenyl]-4-yl)amino)phenyl)-[2,2'-bithiophene]-5-carbaldehyde (17)

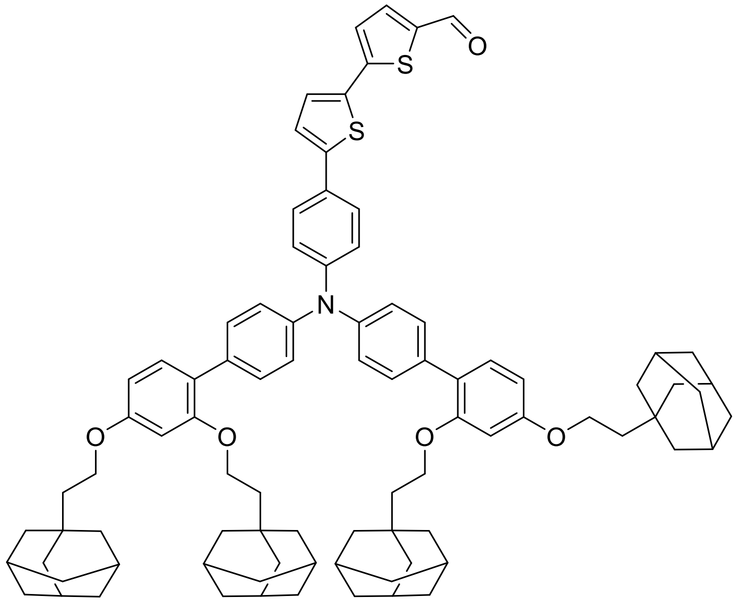

Compound 1**4** (290 mg, 0.24 mmol) was added along with $PdCl_2(CH_3CN)_2$ (2.4 mg, 9 µmol), SPhos (7.5 mg, 18 µmol). The compounds were dissolved in 1,4-dioxane (1 mL), then 4,4,5,5-tetramethyl-1,3,2-dioxaborolane (0.1 mL, 0.69 mmol) and NEt_3 (0.15 mL, 1.08 mmol) were added. The reaction mixture was heated to 110 °C and left stirring for one hour before the reaction mixture was cooled to room temperature. The contents of the reaction were filtered through a Celite plug using EtOAc as eluent, the solvents were then removed in vacuo. The crude boronic ester was a brown viscous oil that was used without further purification.

5′-Bromo-2,2′-bithiophene-5-carboxaldehyde (60 mg, 0.24 mmol), K_2CO_3 (200 mg, 1.5 mmol), $Pd(OAc)_2$ (3.5 mg, 15 µmol), SPhos (12.6 mg, 30 µmol) along with 0.30 g of the crude boronate product were added. 1,4-Dioxane (5 mL) and water (5 mL) were degassed and added under nitrogen, the reaction mixture was stirred at 80 °C for 18 hours. After cooling to room temperature, water (50 mL) was added, and the aqueous phase was extracted with dichloromethane (3 x 40 mL). The combined organic phases were dried over anhydrous Na_2SO_4, filtered and the solvents were removed *in vacuo.* The residue was purified by silica gel column chromatography (dichloromethane/*n*-pentane, 1:8, R_f = 0.67). The product **17** was isolated as a red-orange solid (93 mg, 0.07 mmol, 29%) mp 159-161 °C. ^{1}H NMR (600 MHz, CD_2Cl_2) δ: 9.84 (s, 1H), 7.69 (d, 1H, J = 4.0 Hz), 7.51 (app. d, J = 8.7 Hz, 2H), 7.46 (app. d, J = 8.6 Hz, 4H), 7.36 (d, 1H, J = 3.9 Hz), 7.28 (d, 1H, J = 3.9 Hz), 7.25-7.22 (m, 3H), 7.16-7.12

(m, 6H), 6.55-6.52 (m, 4H), 4.07 (t, 4H, *J* = 7.2 Hz), 4.04 (t, 4H, *J* = 7.2 Hz), 1.99-1.97 (m, 6H), 1.95-1.91 (m, 6H), 1.76-1.64 (m, 24H), 1.64-1.61 (m, 12H), 1.61-1.56 (m, 8H), 1.56-1.54 (m, 12H); ^{13}C NMR (150 MHz, CD_2Cl_2) δ: 182.9, 160.3 (2C), 157.6 (2C), 148.8, 147.6, 146.9, 145.9 (2C), 142.0, 138.1, 134.52, 134.50 (2C), 131.3 (2C), 131.0 (4C), 127.9, 127.3 (2C), 127.1 (2C), 124.8 (4C), 124.4, 123.7(2C, 123.5 (2C), 123.2 (2C), 106.0 (2C), 100.7 (2C), 65.1 (2C), 64.7 (2C), 43.7 (2C), 43.5 (2C), 43.24 (6C), 43.21 (6C), 37.6 (12C), 32.4 (2C), 32.3 (2C), 29.44 (6C), 29.42 (6C); IR (neat, cm^{-1}) ν: 2900 (s), 2846 (m), 1668 (m), 1601, 1493, 1453 (s), 1177, 1048; HRMS (ASAP+, *m/z*): found 1301.6940 (calcd. $C_{87}H_{99}NO_5S_2$ 1301.6965, $[M]^+$).

Synthesis of (*E*)-3-(5'-(4-(bis(2',4'-bis(hexyloxy)-[1,1'-biphenyl]-4-yl)amino)phenyl)-[2,2'-bithiophen]-5-yl)-2-cyanoacrylic acid (JK-305)

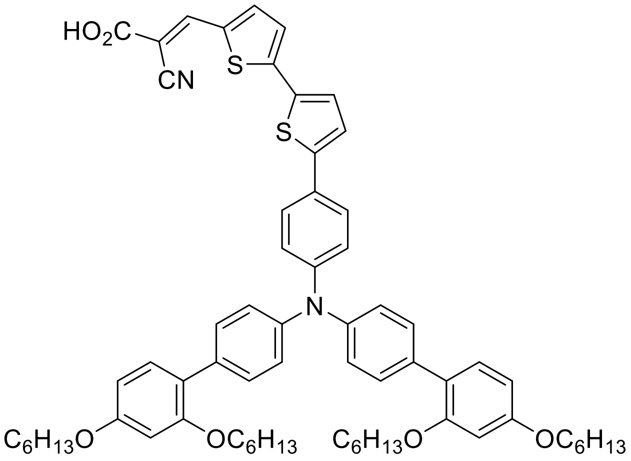

Compound **15** (75 mg, 0.08 mmol) and cyanoacetic acid (130 mg, 1.51 mmol) were dissolved in degassed acetonitrile (22 mL) under nitrogen atmosphere. Piperidine (0.09 mL, 0.9 mmol) was added, and the reaction was heated to 80 °C for 60 minutes before cooling to room temperature and quenched in aqueous HCl (4 M, 50 mL). Dichloromethane (3 × 70 mL) was used to extract the aqueous phase, and the combined organic phases were washed with water (4 × 100 mL), then dried over anhydrous Na_2SO_4, filtered and the solvents were removed *in vacuo*. The crude product was purified by silica gel column chromatography (gradient: 0-15% MeOH in CH_2Cl_2), to obtain sensitizer **BSH-1** as a dark solid (63 mg, 0.06 mmol, 79%), 153-155 °C. ^{1}H NMR (600 MHz, DMSO-d_6, 80 °C) δ: 8.40 (s, 1H), 7.93 (d, *J* = 4.1 Hz, 1H), 7.61 (d, *J* = 9.2 Hz, 2H), 7.56 (d, *J* = 4.9 Hz, 1H), 7.52 (d, *J* = 4.9 Hz, 1H), 7.48-7.42 (m, 5H), 7.23 (d, *J* = 8.4 Hz, 2H), 7.10 (d, *J* = 8.5 Hz, 4H), 7.06 (d, *J* = 9.2 Hz, 2H), 6.62 (d, *J* = 2.8 Hz, 2H), 6.58 (dd, *J* = 2.8, 8.4 Hz, 2H), 4.01 (t, *J* = 6.7 Hz, 4H), 3.98 (t, *J* = 6.4 Hz, 4H), 1.74 (q, *J* = 6.7 Hz, 4H), 1.66 (q, *J* = 6.4 Hz, 4H), 1.48-1.42 (m, 4H), 1.39-1.32 (m, 12H), 1.29-1.23 (m, 8H), 0.90 (t, *J* = 6.7 Hz, 6H), 0.83 (t, *J* = 6.4 Hz, 6H) ($CO_2\underline{H}$ proton missing); ^{13}C NMR (150 MHz, DMSO-d_6, 80 °C) δ: 163.0, 159.0 (2C), 156.4 (2C), 147.3, 145.4, 145.3, 145.2, 144.4 (2C), 140.1, 133.5, 133.3 (2C), 132.9, 130.1 (2C), 129.7 (4C), 127.7, 126.23 (2C), 126.16, 124.3, 123.8, 123.4 (4C), 122.2 (2C), 122.0 (2C), 116.0, 106.2 (2C), 100.6 (2C), 98.2, 67.9 (2C), 67.5 (2C), 30.5 (2C), 30.4 (2C), 28.3 (2C), 28.2 (2C), 24.74 (2C), 24.72 (2C), 21.50 (2C), 21.47 (2C), 13.3 (2C), 13.2 (2C); IR (neat, cm^{-1}) ν: 3411 (br w), 2927 (m), 2856 (m), 1658 (m), 1606 (m), 1492 (m), 1446 (s), 1272 (m), 1023 (m), 1002 (s), 823 (w), 761 (w); HRMS (ASAP+, *m/z*): found 1057.5205 (calcd. $C_{66}H_{77}N_2O_6S_2$ 1057.5223, $[M+H]^+$).

Synthesis of (*E*)-3-(3'-((2-(adamantan-1-yl)ethoxy)methyl)-5'-(4-(bis(2',4'-bis(hexyloxy)-[1,1'-biphenyl]-4-yl)amino)phenyl)-[2,2'-bithiophen]-5-yl)-2-cyanoacrylic acid (BSH-2)

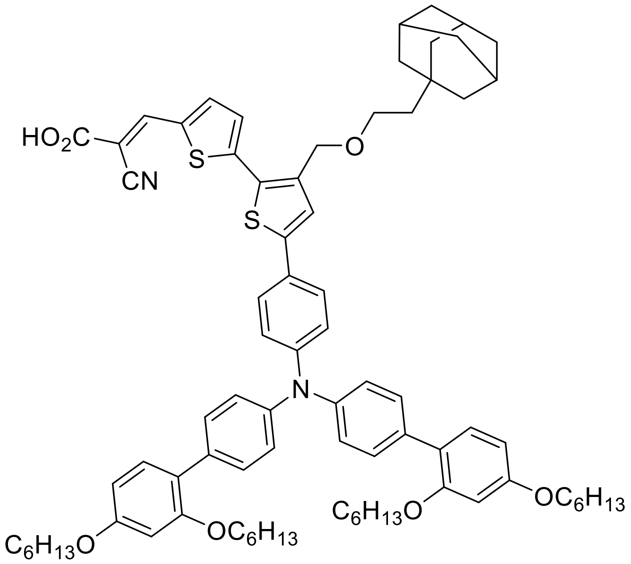

Compound **16** (120 mg, 0.10 mmol) and cyanoacetic acid (170 mg, 2.1 mmol) were dissolved in degassed acetonitrile (45 mL) under nitrogen atmosphere. Piperidine (0.20 mL, 1.2 mmol) was added and the reaction was heated to 80 °C for 1 hour before cooling to room temperature and quenched in aqueous HCl (4 M, 50 mL). Dichloromethane (3 × 70 mL) was used to extract the aqueous phase, and the combined organic phases were washed with water (4 × 100 mL), then dried over anhydrous Na_2SO_4, filtered and the solvents were removed *in vacuo*. The crude product was purified by silica gel column chromatography (gradient: 0-15% MeOH in CH_2Cl_2), to obtain sensitizer **BSH-2** as a dark solid (87 mg, 0.07 mmol, 67%), mp 75-77 °C (dec.).^{1}H NMR (600 MHz, Acetone-d_6) δ: 8.44 (s, 1H), 7.97 (d, J = 4.3 Hz, 1H), 7.60 (d, J = 8.7 Hz, 2H), 7.51 (d, J = 9.0 Hz, 4H), 7.49-7.47 (m, 2H), 7.25 (d, J = 8.8 Hz, 2H), 7.14 (d, J = 9.0 Hz, 4H), 7.11 (d, J = 8.7 Hz, 2H), 6.63 (d, J = 2.3 Hz, 2H), 6.57 (dd, J = 2.3, 8.8 Hz, 2H), 4.61 (s, 2H), 4.03-3.99 (m, 8H), 3.66 (t, J = 7.1 Hz, 2H), 1.92-1.88 (m, 3H), 1.78 (app. q, J = 8.1 Hz, 4H), 1.73 (app. q, J = 7.7 Hz, 4H), 1.71-1.61 (m, 6H), 1.56 (d, J = 2.4 Hz, 6H), 1.52-1.40 (m, 12H), 1.40-1.25 (m, 20H), 0.91 (t, J = 8.1 Hz, 6H), 0.86 (t, J = 7.7 Hz, 6H) ($CO_2\underline{H}$ proton missing); ^{13}C NMR (150 MHz, Acetone-d_6) δ: 163.9, 160.8 (2C), 158.0 (2C), 149.2, 147.2, 146.3, 146.1 (2C), 145.3, 140.89, 140.87, 136.2, 135.1 (2C), 131.6 (2C), 131.3 (4C), 131.2, 127.51, 127.45, 127.4 (2C), 127.2, 125.0 (4C), 123.6 (2C), 123.4 (2C), 116.9, 106.7 (2C), 101.1 (2C), 98.9, 69.0 (2C), 68.6 (2C), 67.4, 67.2, 44.5, 43.4 (3C), 37.8 (3C), 32.5, 32.4 (2C), 32.3 (2C), 29.8 (4C)*, 29.5 (3C)*, 26.6 (2C), 26.5 (2C), 23.3 (4C), 14.4 (2C), 14.3 (2C); IR (neat, cm^{-1}) ν: 3500 (br w), 2902 (m), 2849 (m), 2218 (w), 1686 (m), 1599 (m), 1490 (m), 1408 (s), 1262 (m), 1178 (s), 825 (m), 734 (s); HRMS (ASAP+, *m/z*): found 1248.6650 (calcd. $C_{79}H_{96}N_2O_7S_2$ 1248.6659, $[M]^+$). *The ^{13}C signals were found using ^{1}H-^{13}C HSQC spectroscopy due to their overlap with the residual solvent signal.

Synthesis of (*E*)-3-(5'-(4-(bis(2',4'-bis(2-(adamantan-1-yl)ethoxy)-[1,1'-biphenyl]-4-yl)amino)phenyl)-[2,2'-bithiophen]-5-yl)-2-cyanoacrylic acid (BSH-3)

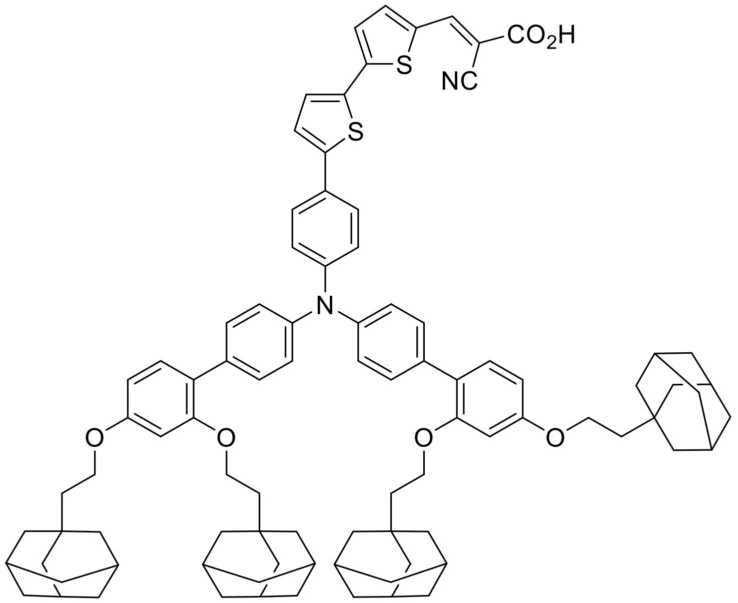

Compound **17** (66 mg, 0.05 mmol) and cyanoacetic acid (90 mg, 1.1 mmol) were dissolved in degassed acetonitrile (40 mL) under nitrogen atmosphere additionally chloroform (5 ml) was added to dissolve **17**. Piperidine (0.10 mL, 0.64 mmol) was added, and the reaction was heated to 80 °C for 3 hours before cooling to room temperature and quenched in aqueous HCl (4 M, 50 mL). Dichloromethane (3 × 70 mL) was used to extract the aqueous phase, and the combined organic phases were washed with water (4 × 100 mL), then dried over anhydrous Na_2SO_4, filtered and the solvents were removed *in vacuo*. The crude product was purified by silica gel column chromatography (gradient: 0-15% MeOH in CH_2Cl_2), to obtain sensitizer **BSH-3** as a dark purple solid (39 mg, 0.03 mmol, 57%), 206-207 °C (dec.). ^{1}H NMR (600 MHz, THF-d_8) δ: 8.32 (s, 1H), 7.79 (d, *J* = 4.1 Hz, 1H), 7.56 (app. d, *J* = 8.7 Hz, 2H), 7.48 (d, *J* = 3,9 Hz, 1H), 7.44 (app. d, *J* = 8.6 Hz, 4H), 7.38 (d, *J* = 4,0 Hz, 1H), 7.34 (d, *J* = 3,9 Hz, 1H), 7.20 (d, *J* = 8.4 Hz, 2H), 7.14-7.11 (m, 6H), 6.56 (d, *J* = 2.3 Hz, 2H), 6.53 (dd, *J* = 2.3, 8.4 Hz, 2H), 4.08-4.02 (m, 8H), 1.99-1.95 (m, 6H), 1.94-1.90 (m, 6H), 1.78-1.67 (m, 24H), 1.66-1.64 (m, 12H), 1.61-1.58 (m, 4H), 1.56-1.54 (m, 16H) ($CO_2\underline{H}$ proton missing); ^{13}C NMR (150 MHz, THF-d_8) δ: 164.0, 160.7 (2C), 158.0 (2C), 149.3, 147.6, 147.3, 146.4, 146.3 (2C), 140.3, 135.3, 135.2 (2C), 134.7, 131.4 (2C), 131.2 (4C), 128.5, 127.9 (2C), 127.3, 124.9, 124.8 (4C), 124.4, 123.8 (2C), 123.8 (2C), 116.6, 106.1 (2C), 101.1 (2C), 99.4, 65.1 (2C), 64.2 (2C), 44.2 (2C), 43.9 (2C), 43.6 (6C), 43.5 (6C), 38.0 (12C), 32.7 (2C), 32.6 (2C), 29.88 (6C), 29.87 (6C); IR (neat, cm^{-1}) ν: 2896 (s), 2844 (m), 2217 (w), 1681 (m), 1578 (m), 1491 (m), 1436 (s), 1264 (s), 1181 (m), 1052 (m), 795 (m), 735 (s); HRMS (ASAP+, *m/z*): found 1324.7125 (calcd. $C_{89}H_{100}N_2O_4S_2$ 1324.7125, $[M-CO_2]^+$).

NMR

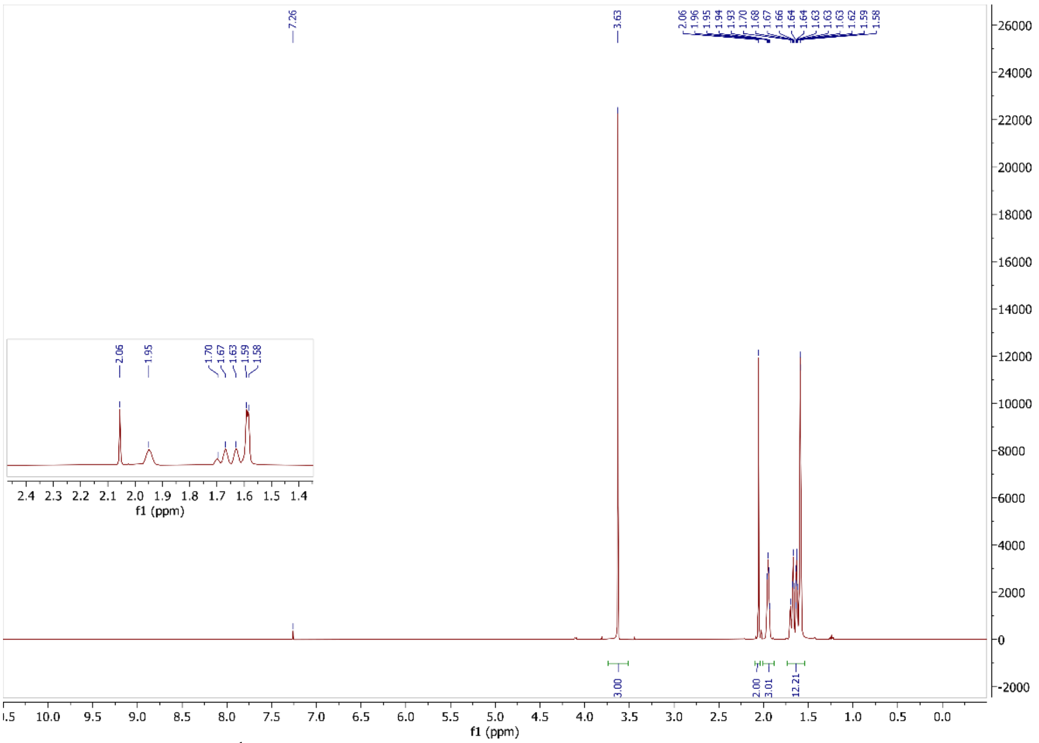

Figure S5. ^{1}H NMR (400 MHz, $CDCl_3$) spectrum for compound **1**.

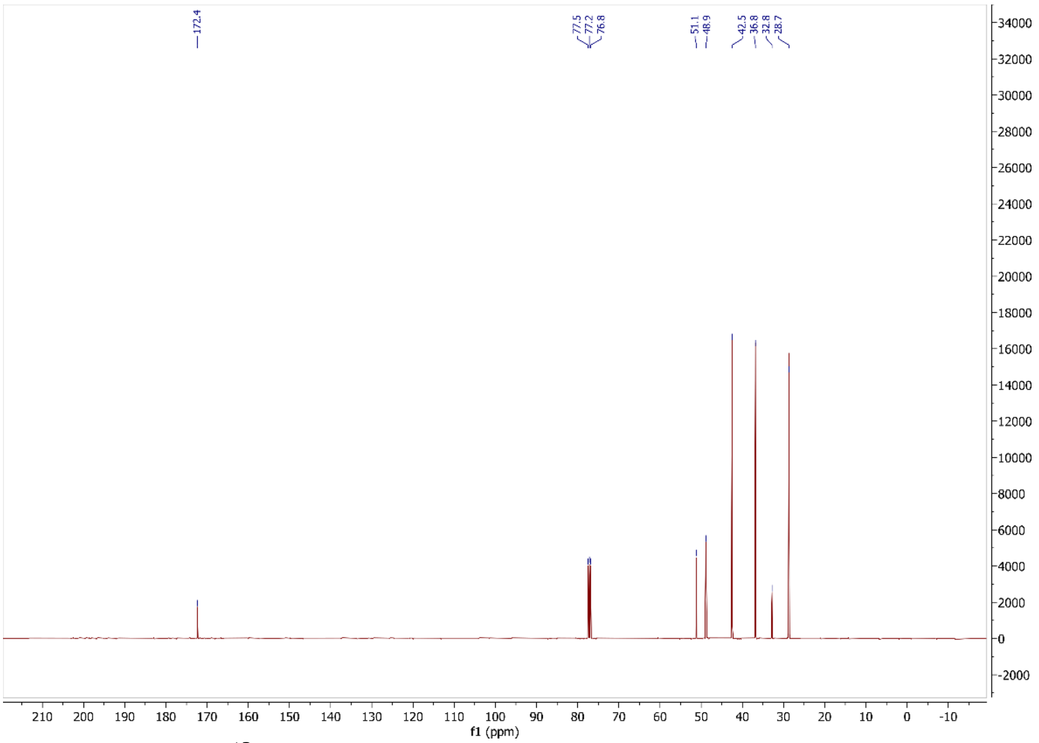

Figure S6. ^{13}C NMR (100 MHz, $CDCl_3$) spectrum for compound **1**.

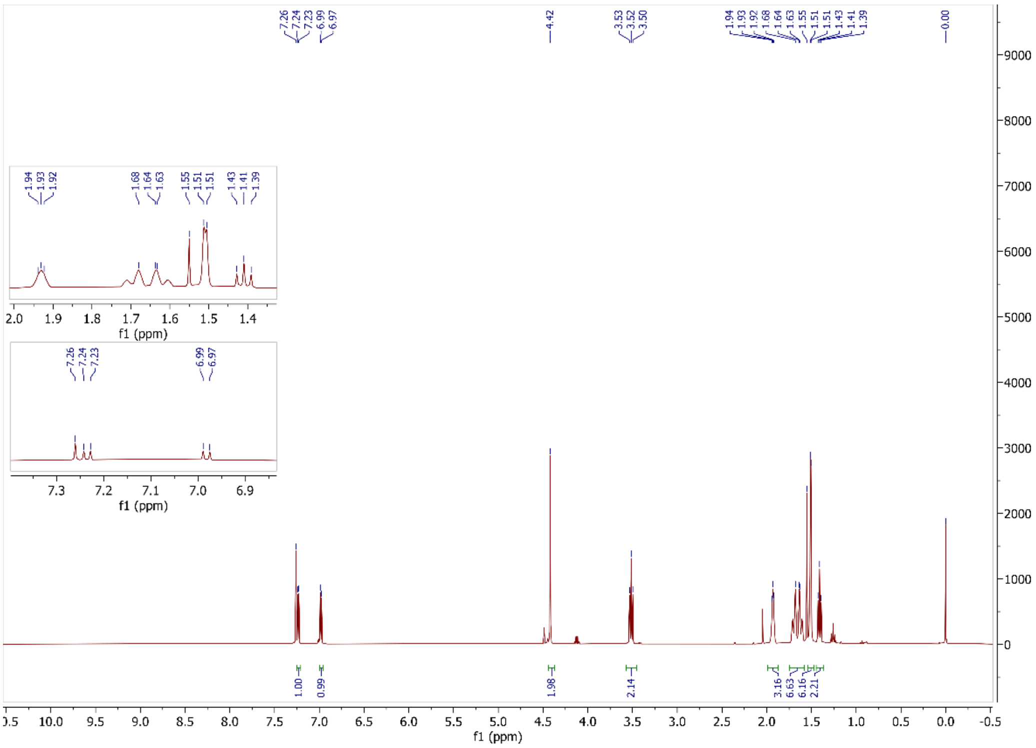

Figure S7. ^{1}H NMR (400 MHz, $CDCl_3$) spectrum for compound **4**.

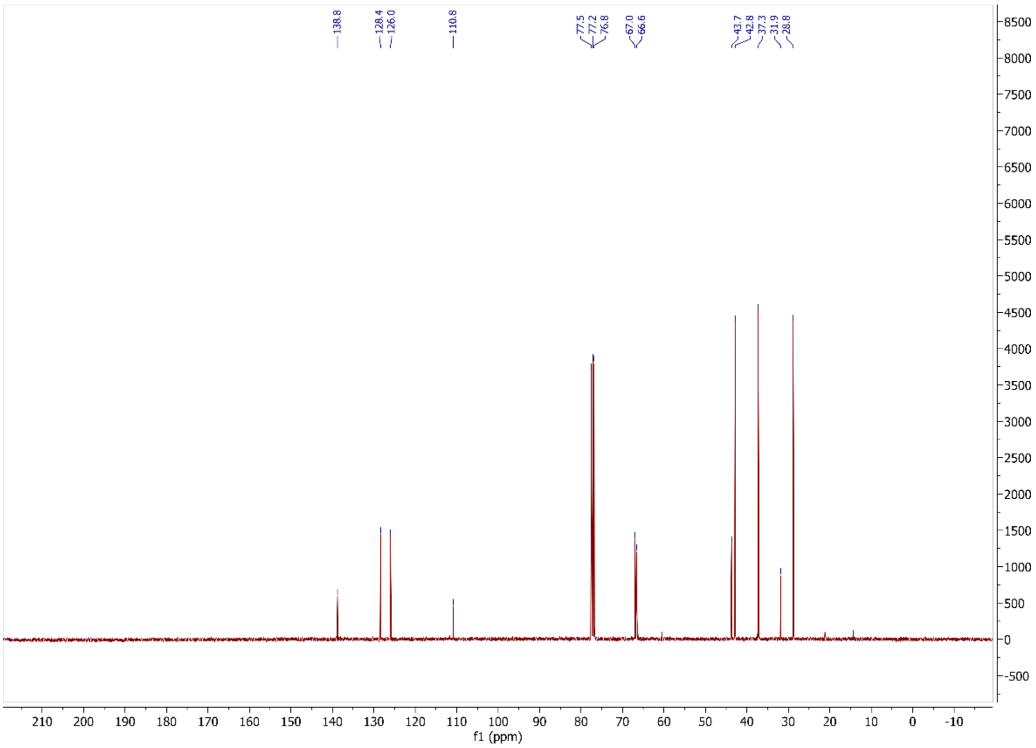

Figure S8. ^{13}C NMR (100 MHz, $CDCl_3$) spectrum for compound **4**.

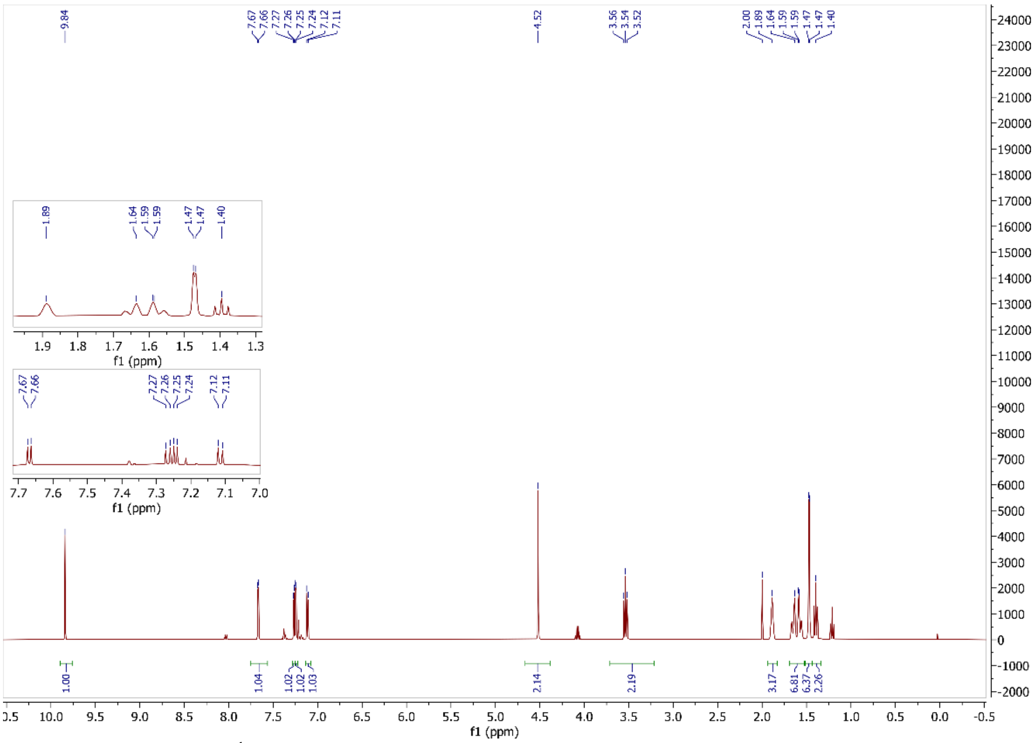

Figure S9. ^{1}H NMR (400 MHz, $CDCl_3$) spectrum for compound **5**.

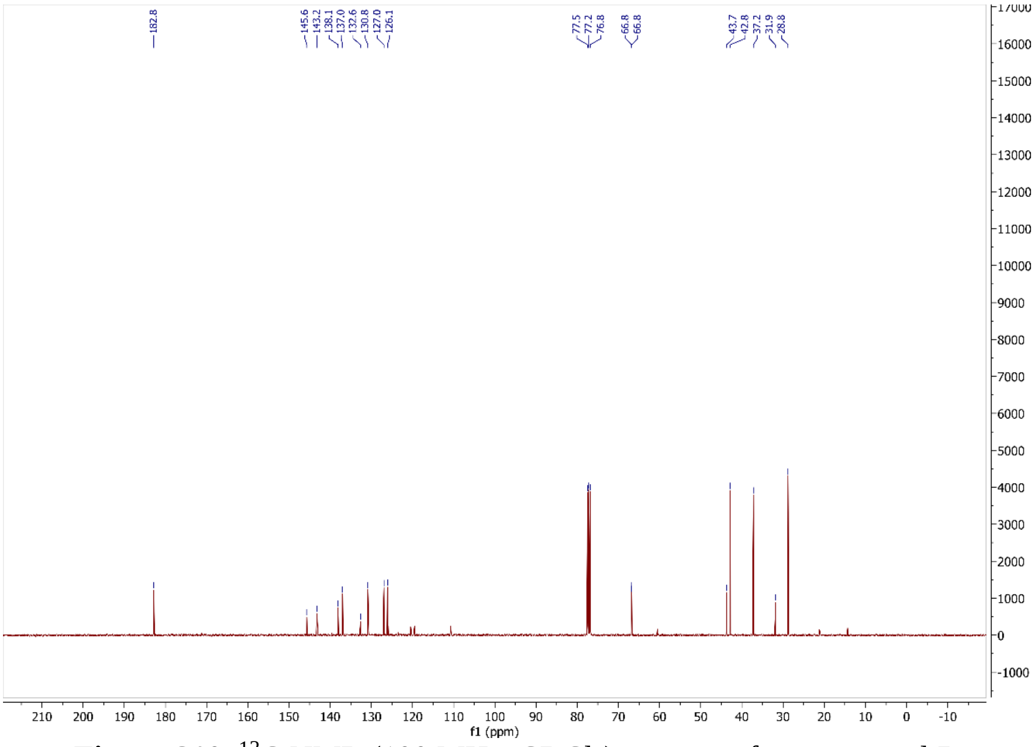

Figure S10. ^{13}C NMR (100 MHz, $CDCl_3$) spectrum for compound **5**.

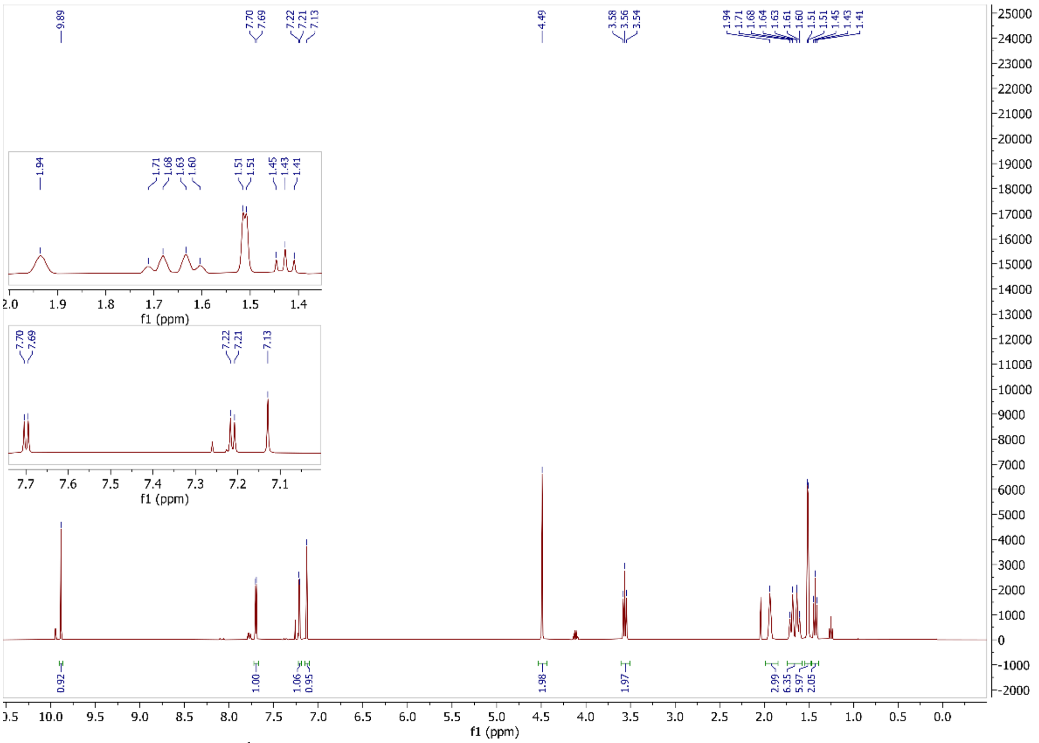

Figure S11. ^{1}H NMR (400 MHz, $CDCl_3$) spectrum for compound **6**.

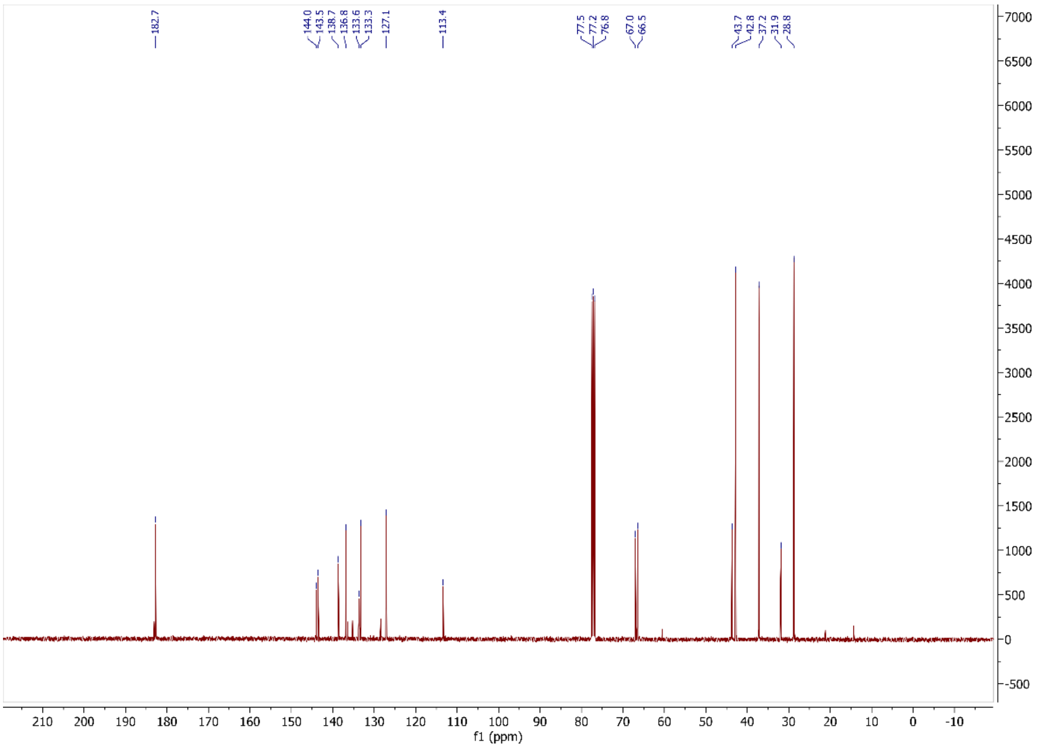

Figure S12. ^{13}C NMR (100 MHz, $CDCl_3$) spectrum for compound **6**.

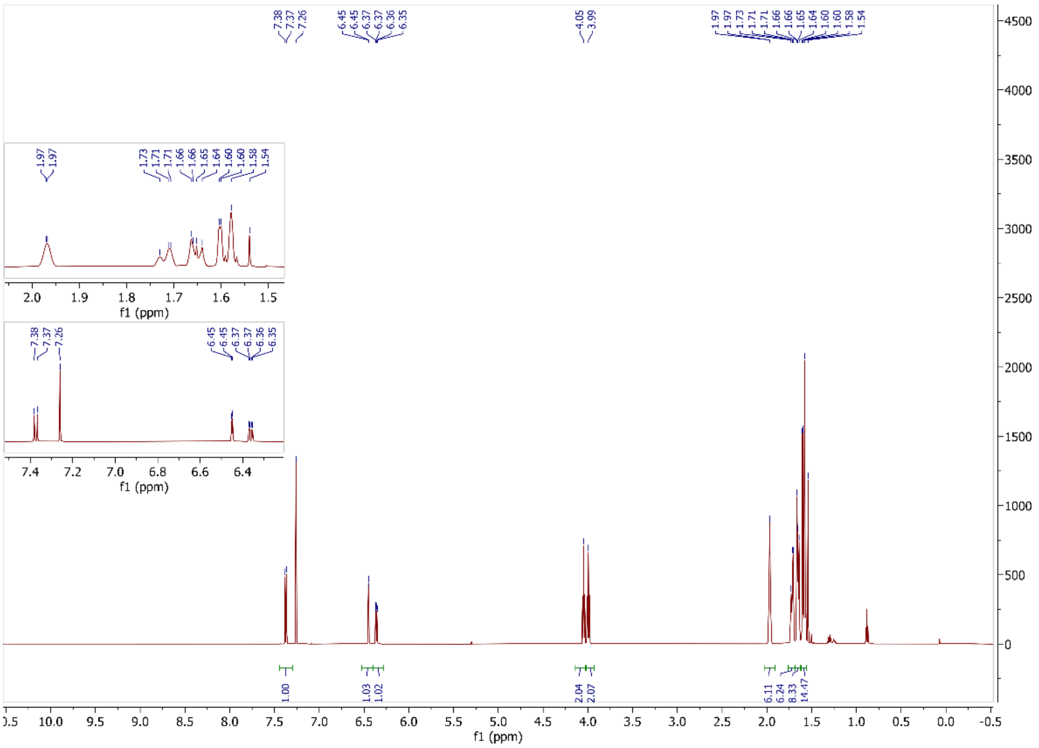

Figure S13. ^{1}H NMR (600 MHz, $CDCl_3$) spectrum for compound **9**.

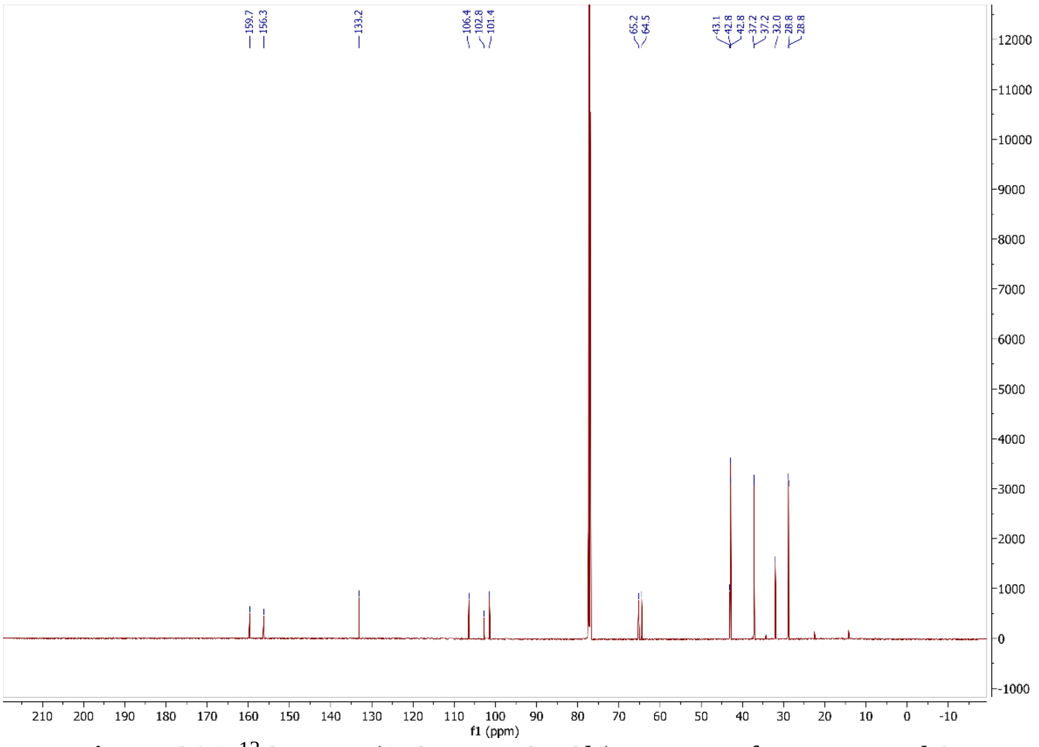

Figure S14. ^{13}C NMR (150 MHz, $CDCl_3$) spectrum for compound **9**.

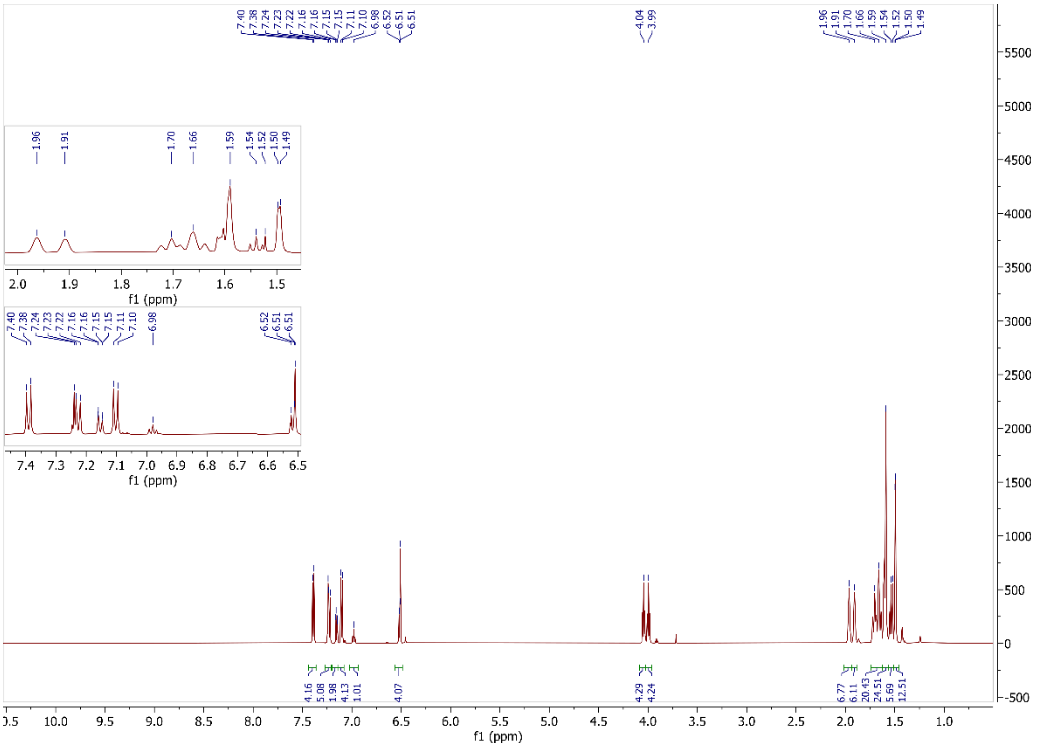

Figure S15. ^{1}H NMR (600 MHz, $CDCl_3$) spectrum for compound **12**.

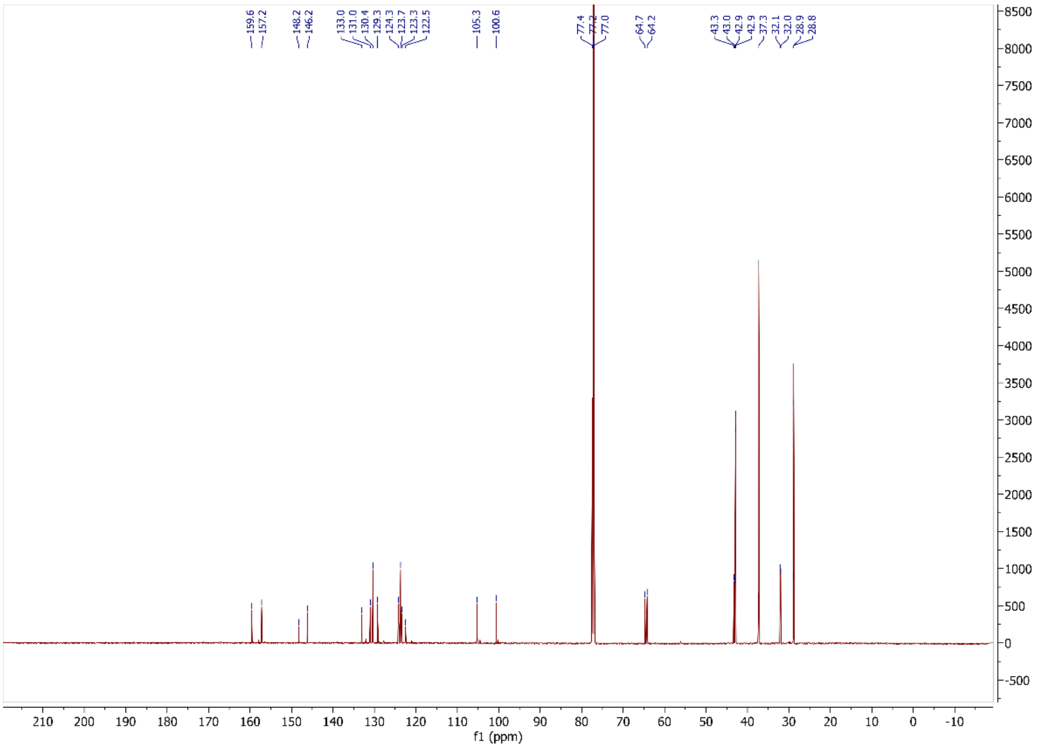

Figure S16. ^{13}C NMR (150 MHz, $CDCl_3$) spectrum for compound **12**.

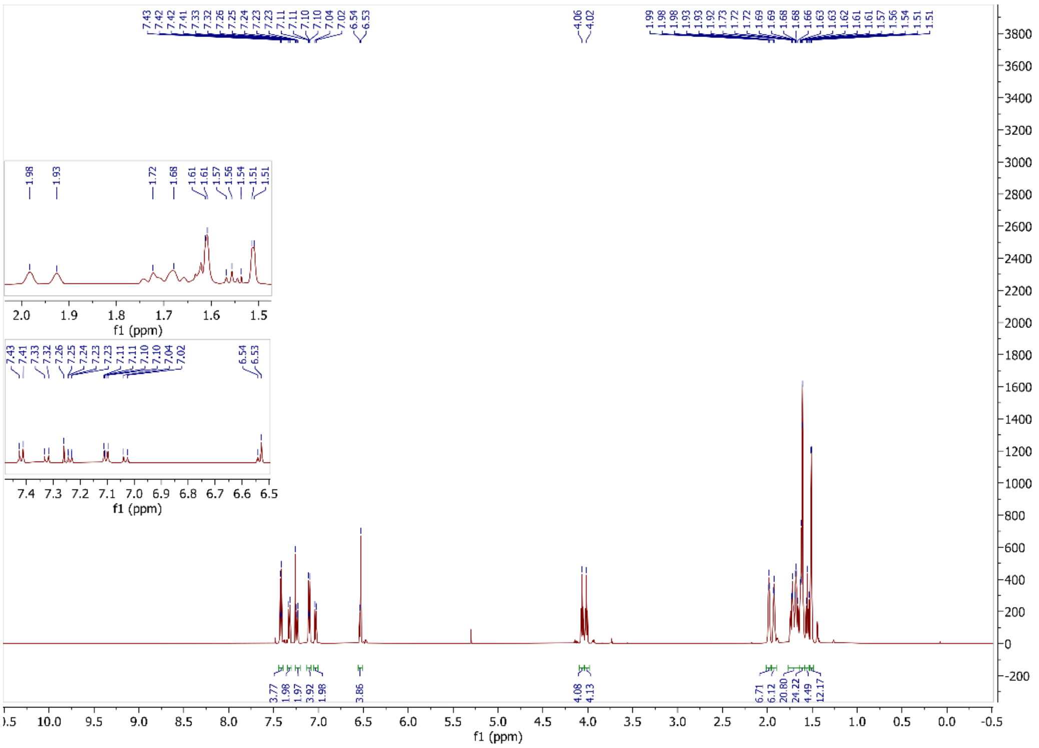

Figure S17. ^{1}H NMR (600 MHz, $CDCl_3$) spectrum for compound **14**.

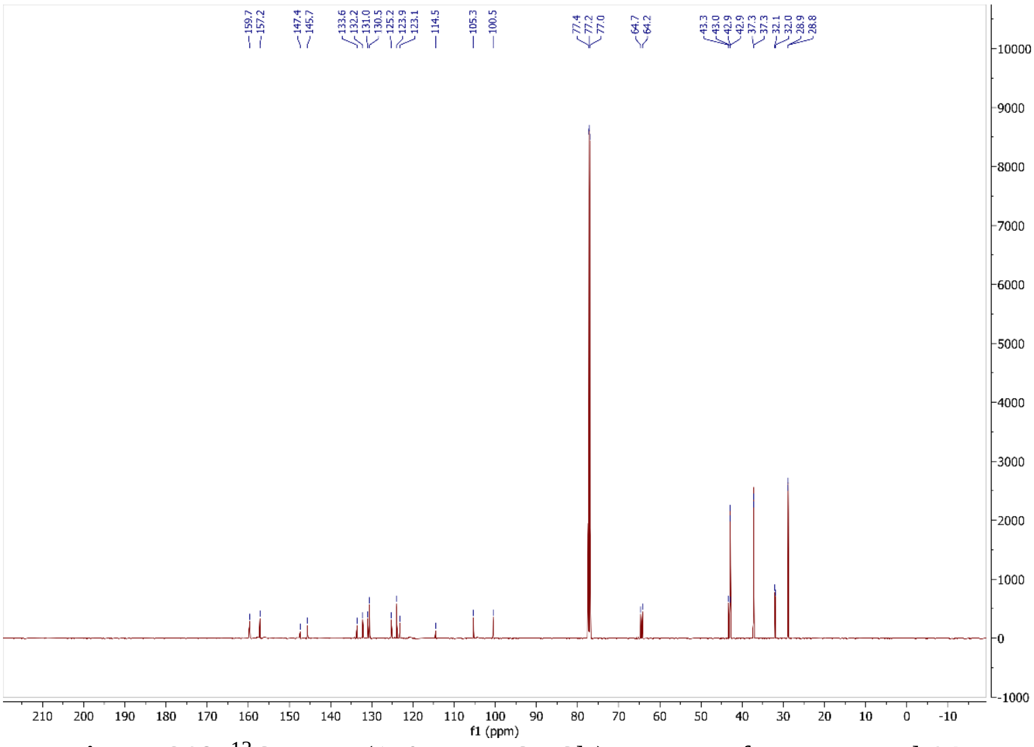

Figure S18. ^{13}C NMR (150 MHz, $CDCl_3$) spectrum for compound **14**.

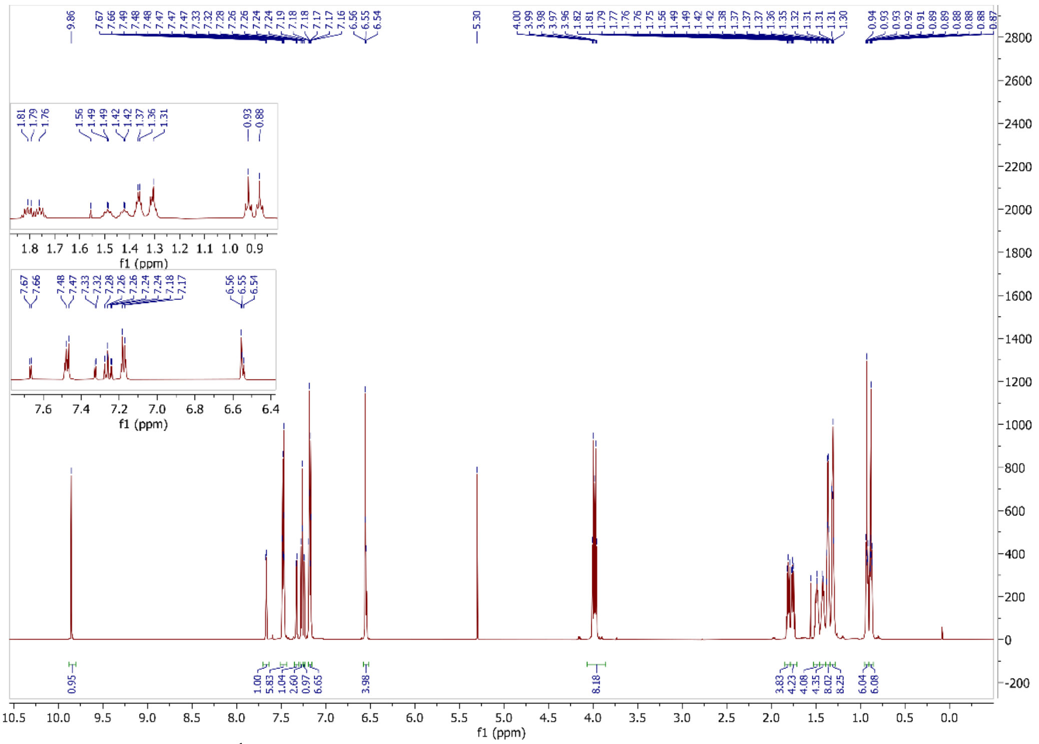

Figure S19. ^{1}H NMR (600 MHz, $CDCl_3$) spectrum for compound **15**.

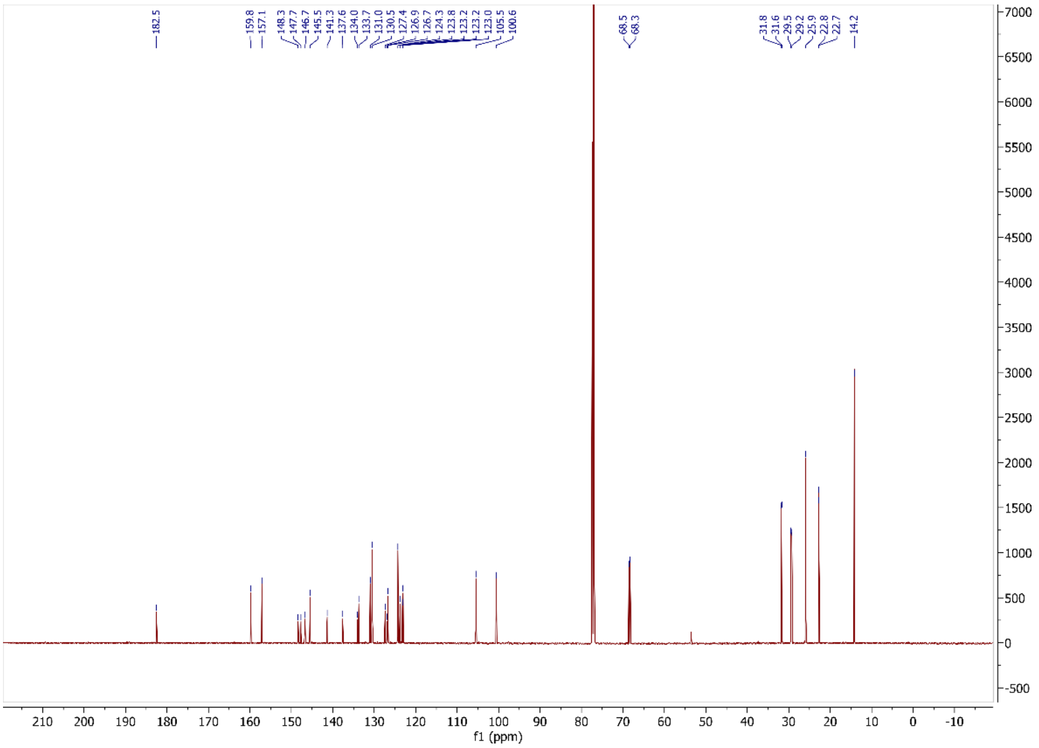

Figure S20. ^{13}C NMR (150 MHz, $CDCl_3$) spectrum for compound **15**.

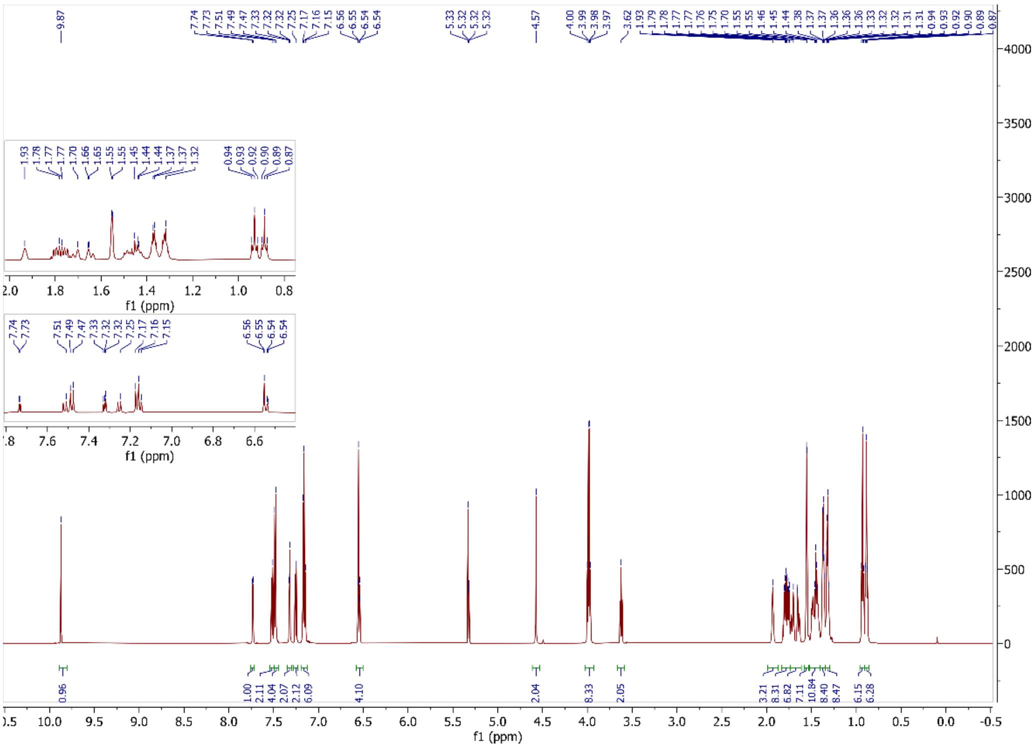

Figure S21. ^{1}H NMR (600 MHz, CD_2Cl_2) spectrum for compound **16**.

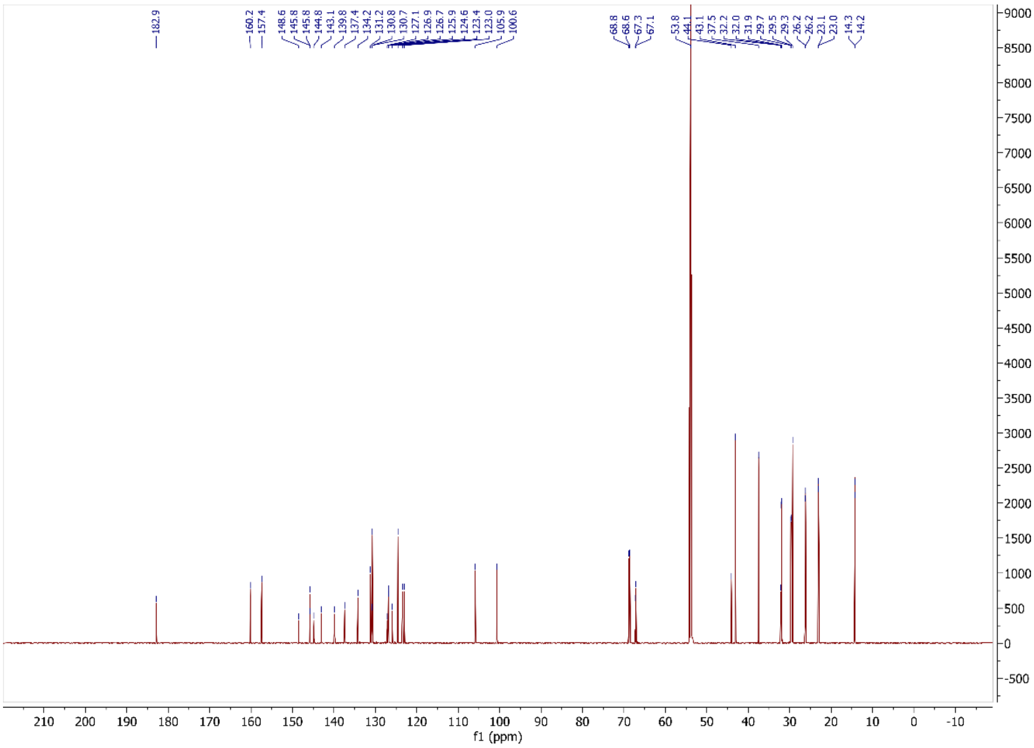

Figure S22. ^{13}C NMR (150 MHz, CD_2Cl_2) spectrum for compound **16**.

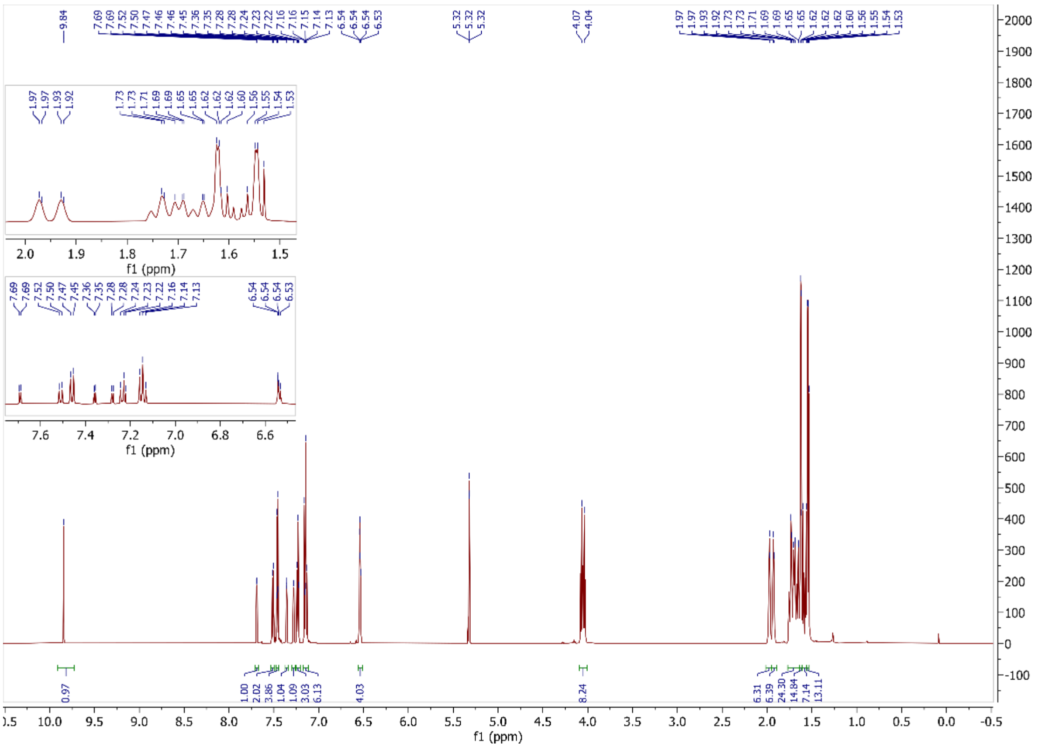

Figure S23. ^{1}H NMR (600 MHz, CD_2Cl_2) spectrum for compound **17**.

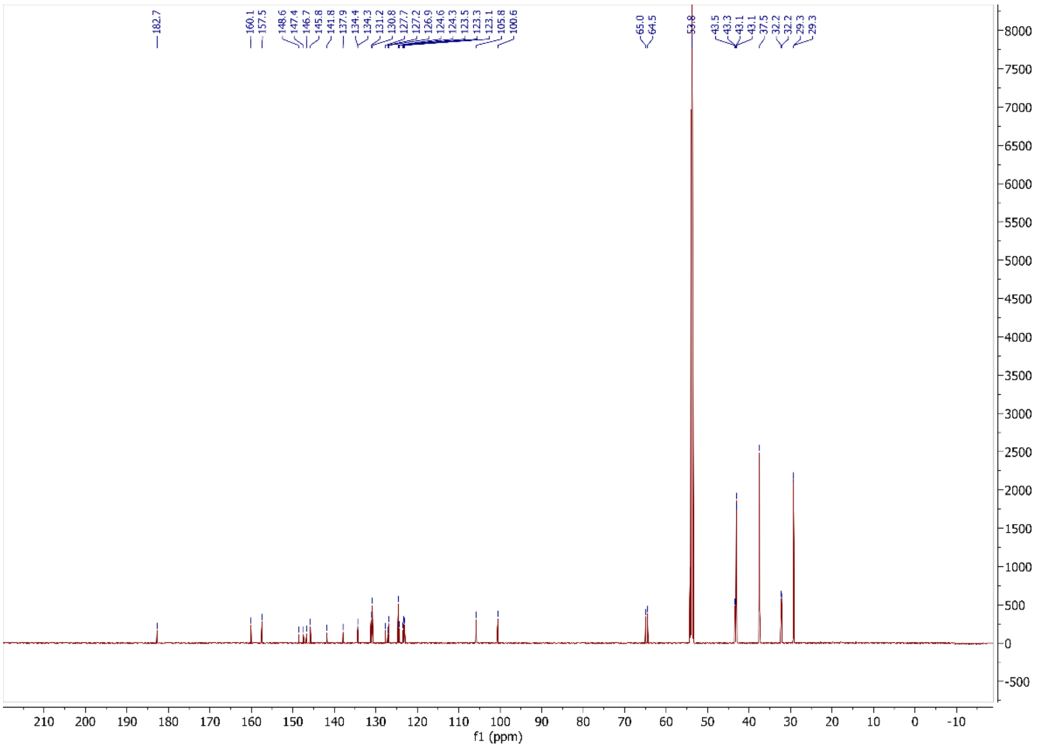

Figure S24. ^{13}C NMR (150 MHz, CD_2Cl_2) spectrum for compound **17**.

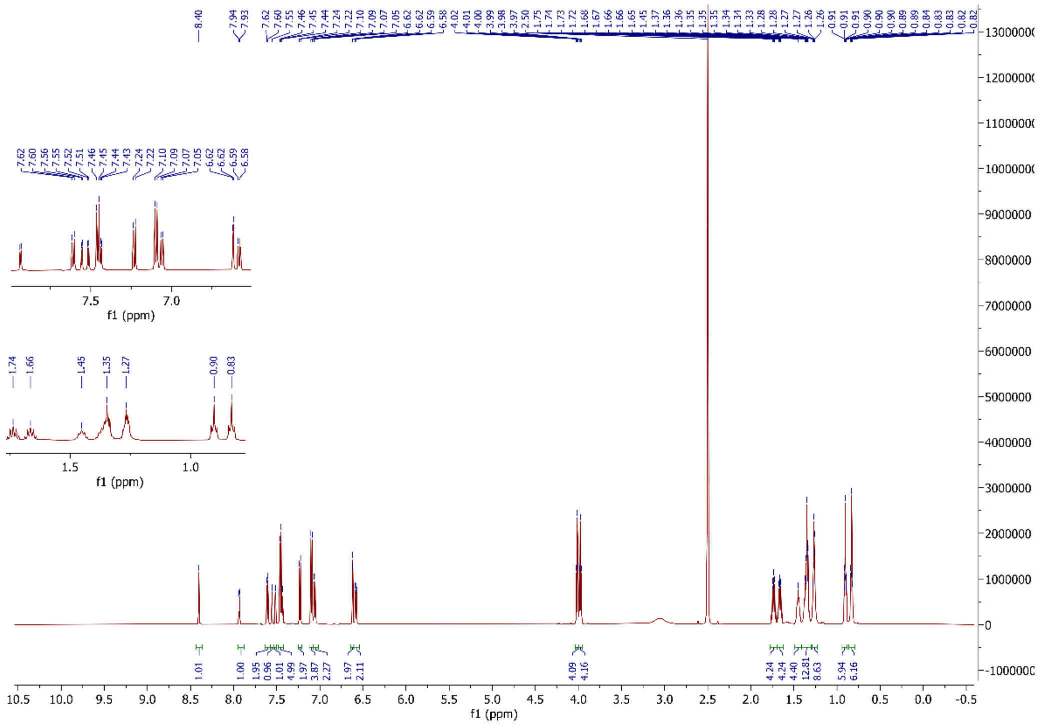

Figure S25. ^{1}H NMR (600 MHz, DMSO-d_6, 80 °C) spectrum for dye **JK-305**.

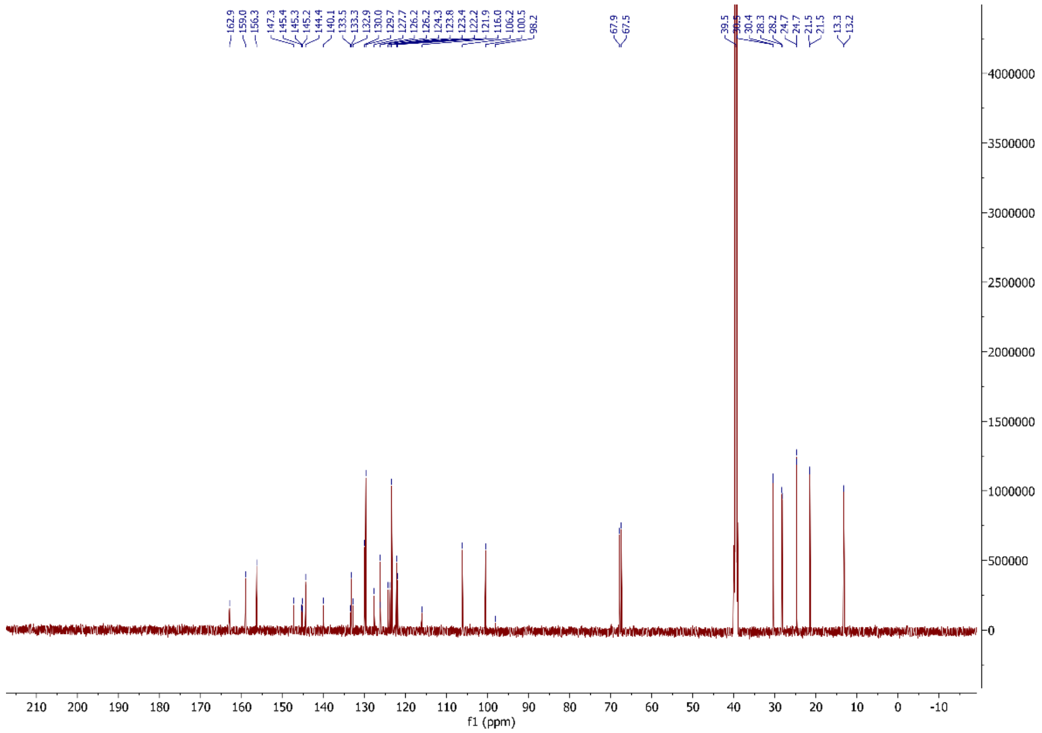

Figure S26. ^{13}C NMR (150 MHz, DMSO-d_6, 80 °C) spectrum for dye **JK-305**.

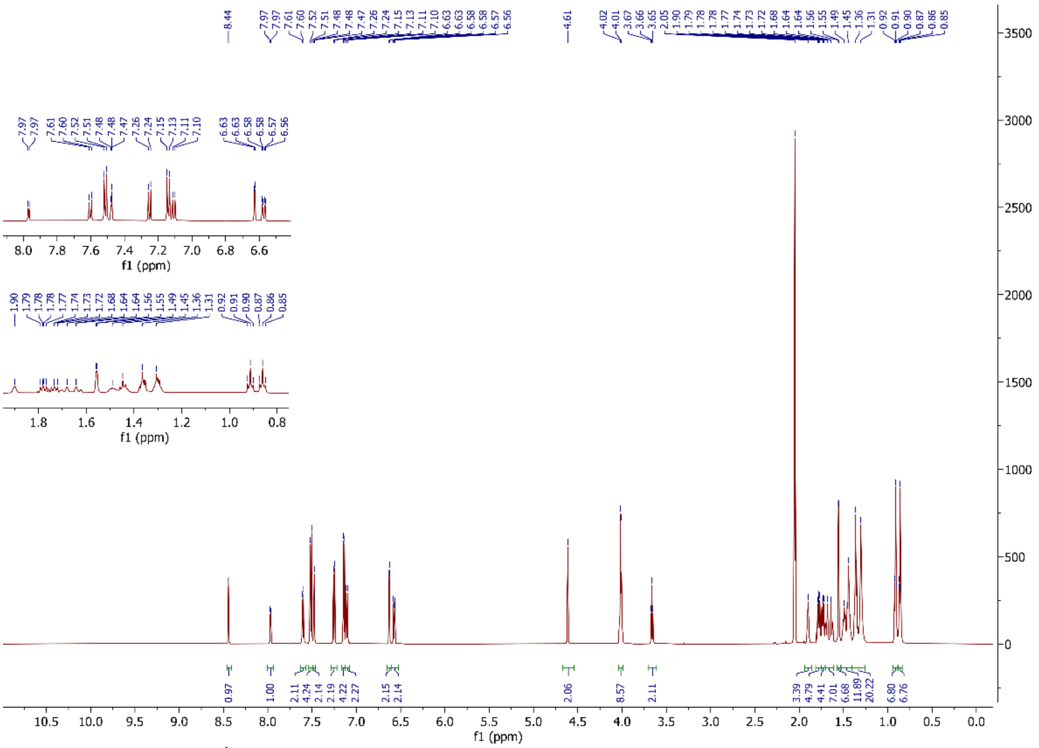

Figure S27. ^{1}H NMR (600 MHz, Acetone-d_6) spectrum for dye **BSH-2**.

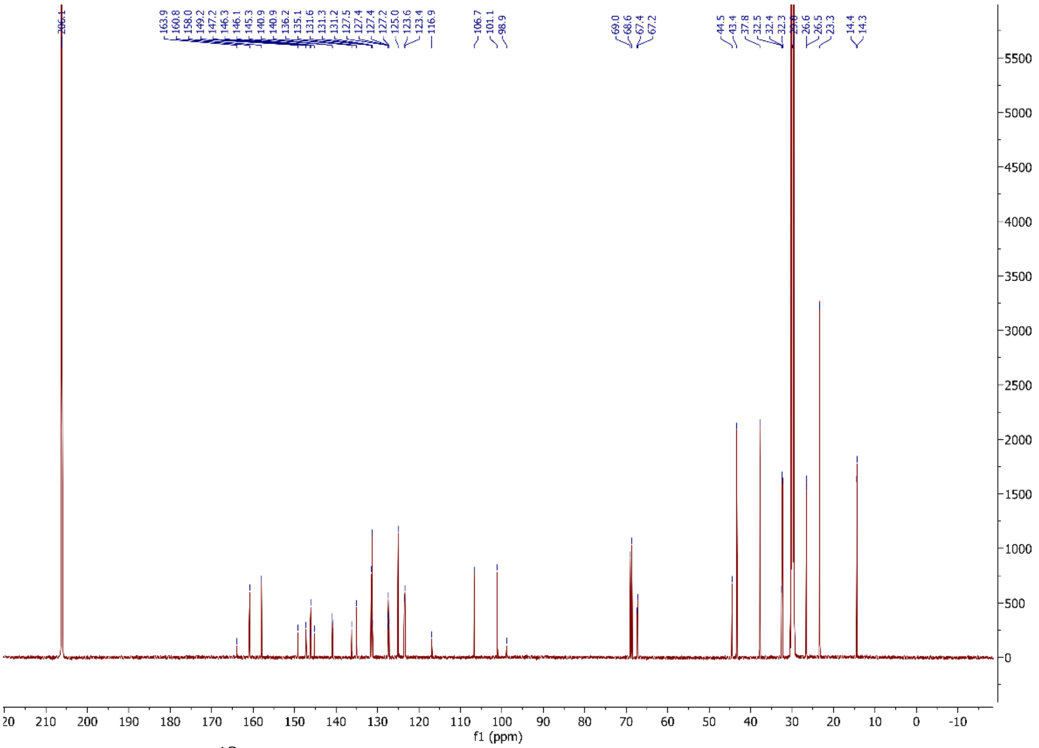

Figure S28. ^{13}C NMR (150 MHz, Acetone-d_6) spectrum for dye **BSH-2**.

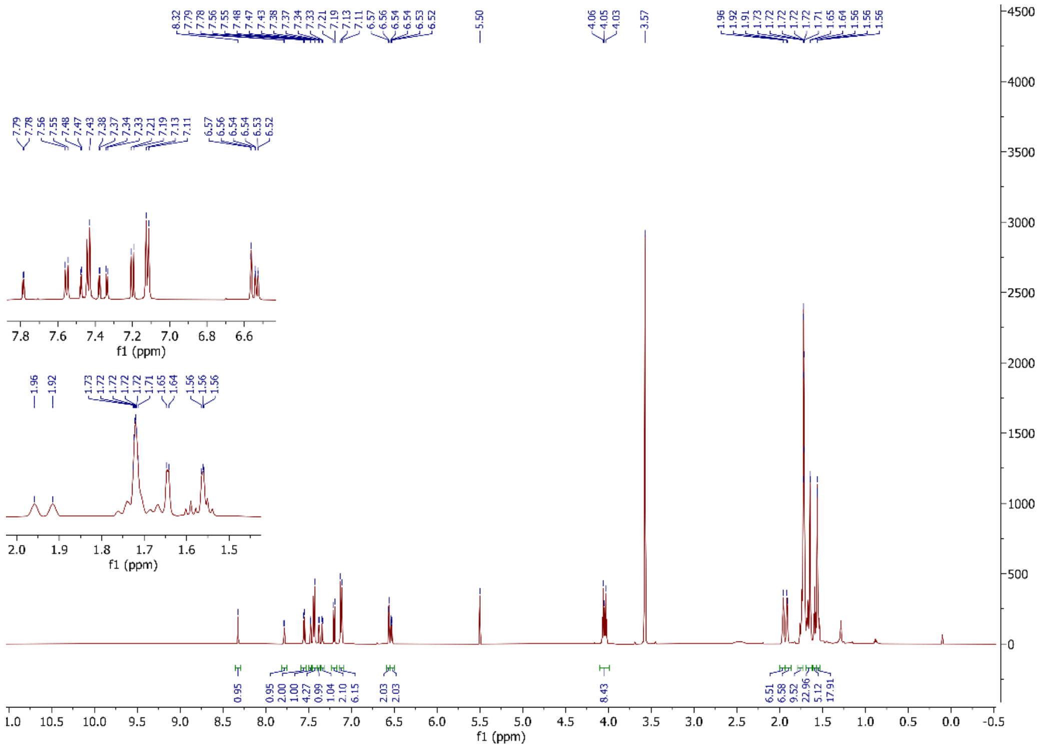

Figure S29. ^{1}H NMR (600 MHz, THF-d_8) spectrum for dye **BSH-3**.

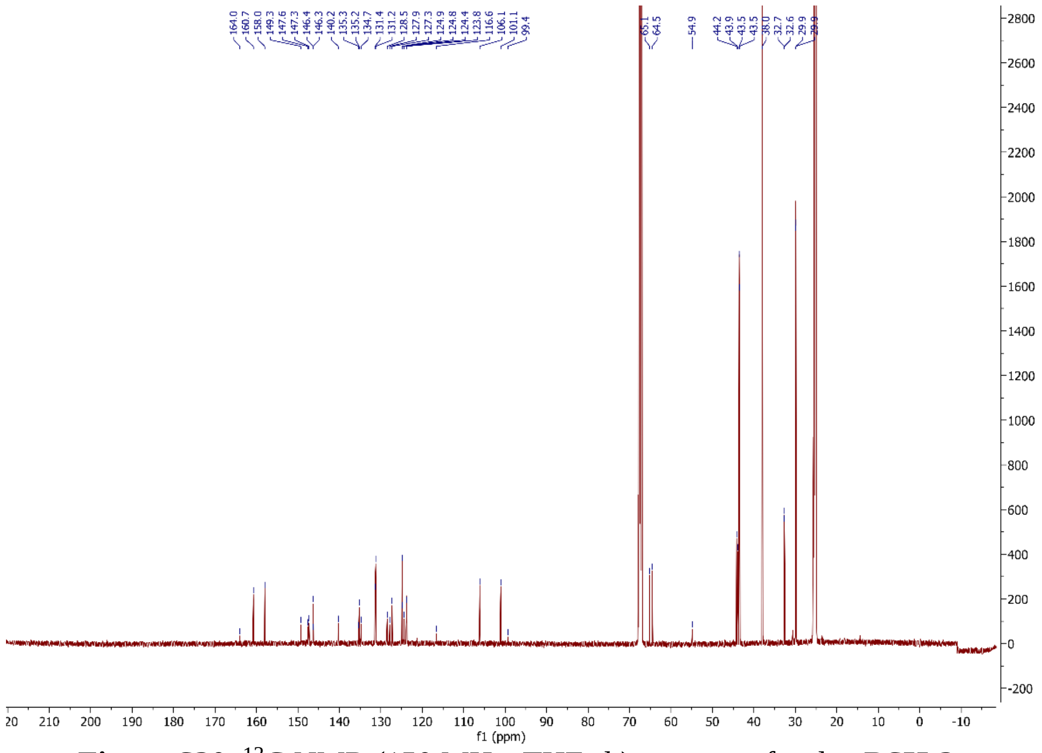

Figure S30. ^{13}C NMR (150 MHz, THF-d_8) spectrum for dye **BSH-3**.

References

[1] Pavlishchuk VV, Addison AW, *Inorg Chim Acta*, **2000**, *298*, 97.
[2] Tsao HN, Yi C, Moehl T, Yum J-H, Zakeeruddin SM, Nazeeruddin MK, Grätzel M, *ChemSusChem*, **2011**, *4*, 591.
[3] Zhang D, Stojanovic M, Ren Y, Cao Y, Eickemeyer FT, Socie E, Vlachopoulos N, Moser J-E, Zakeeruddin SM, Hagfeldt A, Grätzel M, *Nature Communications*, **2021**, *12*, 1777.
[4] Cloutier M, Mamone M, Paquin J-F, *Chem Commun*, **2020**, *56*, 5969.
[5] Belser T, Stöhr M, Pfaltz A, *J. Am. Chem. Soc.*, **2005**, *127*, 8720.
[6] Buene AF, Almenningen DM, Hagfeldt A, Gautun OR, Hoff BH, *Sol. RRL*, **2020**, *4*, 1900569.
[7] Lim, K, Ju, MJ, Song, J, Choi, IT, Do, K, Choi, H, Song, K, Kim, HK, Ko, J, *ChemSusChem*, **2013**, *6*, 1425.
